# Distinct Roles for Arp2/3 Regulators in Actin Assembly and Endocytosis

**DOI:** 10.1371/journal.pbio.0060001

**Published:** 2008-01-03

**Authors:** Brian J Galletta, Dennis Y Chuang, John A Cooper

**Affiliations:** Department of Cell Biology and Physiology, Washington University School of Medicine, St. Louis, Missouri, United States of America; Harvard University, United States of America

## Abstract

The Arp2/3 complex is essential for actin assembly and motility in many cell processes, and a large number of proteins have been found to bind and regulate it in vitro. A critical challenge is to understand the actions of these proteins in cells, especially in settings where multiple regulators are present. In a systematic study of the sequential multicomponent actin assembly processes that accompany endocytosis in yeast, we examined and compared the roles of WASp, two type-I myosins, and two other Arp2/3 activators, along with that of coronin, which is a proposed inhibitor of Arp2/3. Quantitative analysis of high-speed fluorescence imaging revealed individual functions for the regulators, manifested in part by novel phenotypes. We conclude that Arp2/3 regulators have distinct and overlapping roles in the processes of actin assembly that drive endocytosis in yeast. The formation of the endocytic actin patch, the creation of the endocytic vesicle, and the movement of the vesicle into the cytoplasm display distinct dependencies on different Arp2/3 regulators. Knowledge of these roles provides insight into the in vivo relevance of the dendritic nucleation model for actin assembly.

## Author Summary

A branched network of growing actin filaments, pushing against a membrane, provides the force for certain cellular movements. The Arp2/3 complex plays a central role in this process by generating new filaments and branch points. A number of proteins bind to and, in some cases, regulate Arp2/3. It is important to determine, in the cell, the precise roles of each of the many Arp2/3 regulators in generating actin networks during a complex, multistep, cellular movement. In yeast, endocytosis occurs at the plasma membrane in association with the assembly and movement of cortical actin patches, which contain six Arp2/3 regulators. We have used the actin patch as a model system to determine the specific roles of these regulators during patch assembly and movement. We used high-speed video microscopy, coupled with computer-aided particle tracking, to monitor the movement of fluorescently labeled actin patches in cells with one or more mutations of the Arp2/3 regulators. The sensitivity of this technique allowed us to identify previously unappreciated functions for Arp2/3 regulators and to assign each of the regulators a specific role during actin patch assembly and movement. Our results demonstrate that Arp2/3 regulatory proteins play overlapping roles at certain stages of actin patch movement, but distinct roles at other stages. In addition, our results provide new insight into how the assembly of an actin filament networks powers the movement of endocytic vesicles away from the membrane.

## Abstract 3

Branched networks of actin filaments, nucleated by the Arp2/3 complex, power many cellular movements. Quantitative analysis of actin patch motility in budding yeast reveals distinct and overlapping roles for Arp2/3 regulators in endocytosis.

## Introduction

Dynamic networks of branched actin filaments are frequently found adjacent to membranes and appear to play a role in many cellular process (reviewed in [1]). The dendritic nucleation model provides a framework to understand how networks of branched actin filaments assemble and generate a pushing force [2–4]. A key step in the formation of a branched actin filament network is the activation of the Arp2/3 complex, which nucleates a new actin filament from the side of an existing filament. This new growing filament pushes against the membrane. Arp2/3 normally exists in an inactive state and requires an activator protein to induce a large conformational change, which allows for nucleation of a new actin filament (reviewed in [1,5,6]). Coronin, an Arp2/3 inhibitor, stabilizes Arp2/3 in the inactive conformation [7].

In some cases, targeting the Arp2/3 complex to a subcellular location—rather than activating it—may be the principal role for an Arp2/3-binding protein (reviewed in [3,8–10]). Of note, with yeast actin as a substrate, the yeast Arp2/3 complex nucleated polymerization rather well in the absence of an activator [8,11]. Activators enhanced the activity of the Arp2/3 complex in these studies, but by a relatively small amount. Thus, yeast Arp2/3 activators may be critical for spatial control of actin assembly by targeting, rather than activating, Arp2/3.

**Figure 1:**
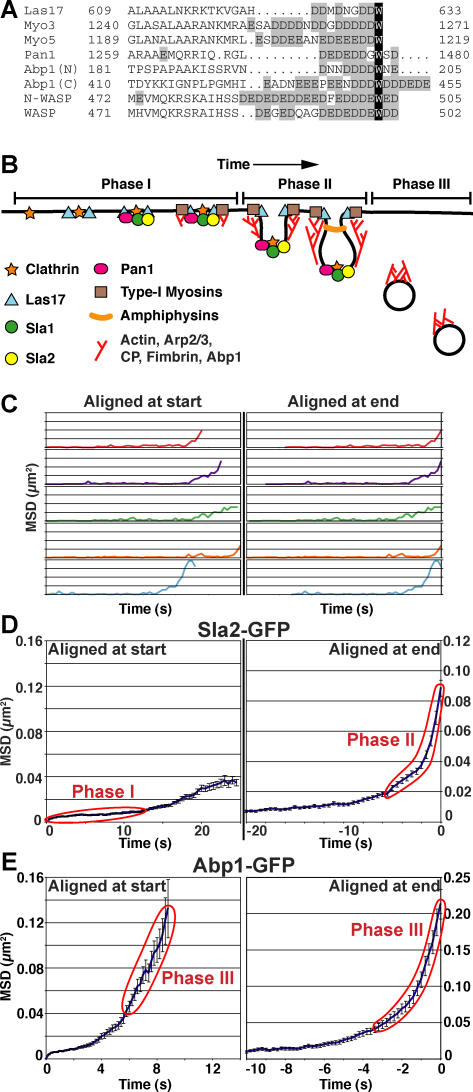
Acidic Domains of Arp2/3 Activators, Model of Actin Patch Assembly and MSD Examples (A) Alignment of acidic/DDW regions of S. cerevisiae Arp2/3 activators with the corresponding regions of human WASp and N-WASp. Acidic residues are highlighted with gray and the conserved tryptophan with black. Numbers are amino acid residues. The entire region shown here was removed in “Δ*acidic*” strains. (B) Model of actin patch formation and movement, adapted from previous studies [23,24,33], are shown. (C) Examples of squared displacement plotted versus time for five individual patches, with the curves aligned at the start (left) or (right) end of patch lifetime. Such curves would then be averaged to generate MSD plots. (D) Typical MSD plots of wild-type patches labeled with Sla2-GFP generated by aligning individual curves at the start (left) or the end (right) of patch lifetime. On the left, the plot is truncated when half of the patches have disappeared, indicating the median lifetime. Phases I and II of patch movement are indicated. (E) Typical MSD plots of wild-type patches labeled with Abp1-GFP generated as in (D). Phase III patch movements are indicated.

An important challenge for the field is to understand how the activities of multiple Arp2/3-activating proteins are coordinated in vivo. Do these activators have overlapping functions or does each of these proteins have a unique role in the formation of a proper network? We addressed this question by investigating the roles of all of the proposed Arp2/3 regulatory proteins in the assembly and movement of cortical actin patches in *Saccharomyces cerevisiae.* Actin patches contain five proteins with an acidic/DDW motif for binding the Arp2/3 complex: a WASp (Las17); two type-I myosins (Myo3 and Myo5); an Eps15 homology (EH) protein (Pan1); and an actin filament–binding protein (Abp1) (Figure 1A) [12]. The acidic/DDW region is necessary and sufficient to bind the Arp2/3 complex, and all of these proteins can bind and activate Arp2/3 in vitro [13–20]. Actin patches also contain a coronin, Crn1, which can inhibit Arp2/3 activation in vitro [21,22]. The actin patch provides an excellent system to test the roles of these regulators in a single, complex, multicomponent system.

In yeast, actin patch assembly and movement mediates endocytosis [23–27]. Actin patches assemble at the plasma membrane as the endocytic vesicle forms. Patches then move into the cell with the vesicle [24,28,29]. Patch formation and movement occurs rapidly, on a time scale of seconds to minutes, and depends on Arp2/3 complex and actin polymerization [29–32].

The life cycle of an actin patch is a stereotyped sequence of events that is characterized by changes in protein composition, location, and movement; this can be seen as three phases (Figure 1B). In phase I, actin patches assemble at the cortex and display a limited amount of motion, as if tethered in place. Many proteins are recruited to the actin patch during this phase, including WASp/Las17 and endocytic adaptors Sla1, Sla2, and Pan1. Near the end of phase I, the actin filament network begins to assemble, as indicated by the arrival of the Arp2/3 complex, capping protein (Cap1/Cap2; CP), and the actin filament–binding proteins fimbrin/Sac6 and Abp1 [24,33]. Phase II is characterized by slow movement of the patch a short distance into the cell, away from the plasma membrane. Some patch components appear to remain at the membrane during this movement, such as Las17 and Myo5 [24,34], whereas others appear to move into the cell, including Sla1, Sla2, Pan1, Arp2/3, CP, and Abp1. At the end of phase II, several proteins are lost from the patch, including Sla1, Sla2, and Pan1. During phase III, patches contain actin filaments and actin-binding proteins, and they undergo more rapid and lengthy movement into the cell before disappearing [24,33].

In this study, we used high-speed video microscopy, coupled with computer-assisted patch tracking and quantitative motion analysis, to study the effect of mutations in genes for Arp2/3 regulators on the assembly and movement of actin patches marked by green fluorescent protein (GFP)-labeled components. The methodology allowed the study of large numbers of patches, which revealed novel phenotypes in the mutants. These studies provide new evidence about the function of each of the Arp2/3 regulatory proteins and reveal that these regulators have some roles that are distinct from each other, as well as some that are overlapping.

## Results

### The Acidic/DDW Domain of WASp/Las17 Is Important for Actin Patch Motility

Las17, the yeast WASp protein, binds to and activates Arp2/3 via an acidic/DDW region at its C terminus (Figure 1A) [11,19]. To determine the role that this region plays in actin patch motility, a C-terminal truncation that removed the acidic/DDW region was generated. All of the mutations described in this study were made at the endogenous locus and were examined in haploids, where the mutant allele was the only allele of the gene present. The mutation removed the Arp2/3-binding region but not other known domains, including the WH2 domain that binds actin monomer. The truncated Las17 localized to actin patches by GFP tagging (data not shown).

**Figure 2:**
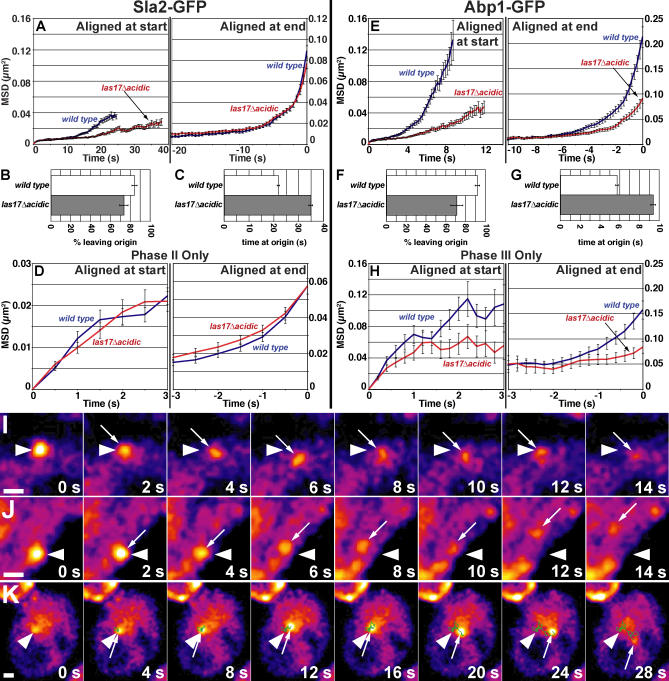
Quantitative Motion Analysis of *las17*Δ*acidic* Patches and the Movement of GFP-WASp/Las17 Particles Cells express Sla2-GFP (A–D), Abp1-GFP (E–H), or GFP-Las17 (I–K). (A) MSD plots for wild-type and mutant patches aligned at the start (left) or end (right) of their lifetimes. The curves on the left are truncated at the median lifetime. (B) Percentage of patches that leave the origin. Mean of values for three segregants is shown. (C) Average time at the origin, from the appearance of a patch until it moved away or disappeared. (D) Phase II movement only. For each patch, data prior to movement were removed. (E) MSD plots for wild-type and mutant patches aligned at the start (left) or end (right) of their lifetimes. On the left, plots are truncated at the median lifetime. (F) Percentage of patches that leave the origin, as in (B). (G) Average time at the origin, as in (C). (H) Phase III movement only. For each patch, data prior to the time the patch traveled 200 nm were removed. Only patches that moved at least 200 nm are included. (I–K) Time-lapse fluorescence images at the indicated intervals. Arrowheads indicate the position of the particle at time zero, and arrows follow the position of the particle over time. (I and J) Particles, presumably actin patches, appear at the cortex and move away. (K) A GFP-WASp/Las17 particle moving inside the cell. The path taken by the particle from time zero is overlaid in green. Scale bars, 500 nm. Strain numbers and the number of patches were as follows: (A–C) Wild-type, YJC5234–6; 106, 93, and 108, respectively. *las17*Δ*acidic*, YJC5231–3; 100, 83, and 73. (D) Strains as in (A–C). Numbers of patches: wild-type – 88, 74, 90. *las17*Δ*acidic* – 66, 67, 53. (E–G) Wild-type, YJC5214–6; 85, 86, and 98 respectively. *las17*Δ*acidic*, YJC5208–10; 95, 76, and 89. (H) Strains as in (E–G). Numbers of patches: wild-type – 76, 69, and 84; *las17*Δ*acidic* – 45, 43, and 49. Error bars are ± standard error. (I–J) YJC5736–7.

We tracked the positions of hundreds of patches over time and then used several forms of quantitative motion analysis to assess the effect of mutations. The methodology is described fully in Materials and Methods. Mean squared displacement (MSD) plots were one form of analysis, and examples of how such plots were generated and used to monitor each phase of the patch life cycle are presented in Figure 1C–1E. We first examined the effects of this mutation on phase I and II of patch motility using Sla2-GFP labeling (Video S1). MSD plots, generated from displacement data of individual patches aligned at the start of their lifetimes and then averaged, indicated a defect in the behavior of *las17*Δ*acidic* patches during phase I or II. In contrast, MSD plots of the same data, averaged after aligning individual patch curves at the end of their lifetimes, showed no defect in the mutant (Figure 2A).

To examine phases I and II more closely, we quantitated directly the frequency and timing of early events. The amount of time that patches spent at their origin, before moving off the membrane, was greatly increased in *las17*Δ*acidic* mutant cells (Figure 2C), which can account for the observed defect in MSD plots aligned at the start (Figure 2A). The percentage of patches that left the membrane, corresponding to transition from phase I to phase II, was essentially normal (Figure 2B). To determine if the timing of the recruitment of actin was normal in *las17*Δ*acidic* cells, we determined the time between the appearance of Sla2 and of Abp1, a marker of actin filaments. The arrival of actin filaments to the patch was delayed in *las17*Δ*acidic* cells (Figure S1). To examine patch movement during phase II, we isolated tracking data for patches—after they moved off the membrane—with Sla2-GFP labeling. In this analysis, the *las17*Δ*acidic* cells were normal (Figure 2D). Taken together, the data support a model where the acidic domain of Las17 is critical for the duration of phase I but not for the ability of the patch to leave the membrane or for its initial movement off the membrane.

The effect of the *las17*Δ*acidic* mutation on the movement of actin patches during phase III was examined using Abp1-GFP (Video S2). Patch movement was decreased in the mutant and was assessed with MSD plots aligned at the start or the end of patch lifetime (Figure 2E). Decreased movement at the start of Abp1-GFP patch life can result from a prolongation of phase I, as seen with Sla2-GFP. Indeed, as expected, the time that Abp1-GFP patches remained at their origin was prolonged (Figure 2G), and the percentage of patches that moved away from their origin was slightly reduced in *las17*Δ*acidic* cells (Figure 2F).

MSD plots aligned at the end of patch lifetimes showed a decrease in movement, as noted above (Figure 2E, right). We tested the phase III movement directly by isolating tracking data for patches after they moved 200 nm from their membrane origin. Patches of *las17*Δ*acidic* cells showed decreased movement in MSD plots aligned at the beginning and end of this movement (Figure 2H).

In previous studies in yeast, GFP-labeled WASp proteins remained on the membrane, exhibiting phase I behavior, and did not move off the membrane [24,33,35] These results raised the question of how the Arp2/3 complex and actin assembly might power the movement of endocytic vesicles into and about the cytoplasm. We found similar results with GFP fused to the C terminus of Las17. However, we find that neither C- nor N-terminal fusions of GFP to Las17, which were expressed from the endogenous *las17* locus, are fully functional for actin patch motility, especially for the late movements of phase III (Figures S2 and S3). Interestingly, when we overexpressed a novel N-terminal fusion from the *GAL1* promoter, approximately one-third of GFP-Las17–labeled patches exhibited substantial movement away from their origin. They moved into and about the cytoplasm, as seen in confocal movies taken at the equator of the cell (Figure 2I–2J, Videos S3–S4). Although this method is technically challenging, we have observed examples of colocalization of these particles moving into the cytoplasm with Abp1-tdimer2 (Video S5). However, this GFP fusion protein may also not reveal the normal localization of the Las17 protein. Actin patch motility in strains expressing this fusion was impaired, especially during phase III (Figure S4), indicating that Las17 function was not normal in these strains. In addition, cells expressing GFP-Las17 from the GAL1 promoter have a distinct population of GFP-Las17 particles moving freely inside the cell (Figure 2K, Video S6). These particles have a markedly greater lifetime than do actin patches, so the particles may correspond to membranous vesicles moving in the cytoplasm reported in previous studies [33,36,37].

### Type-I Myosins Have Distinct but Overlapping Functions

**Figure 3:**
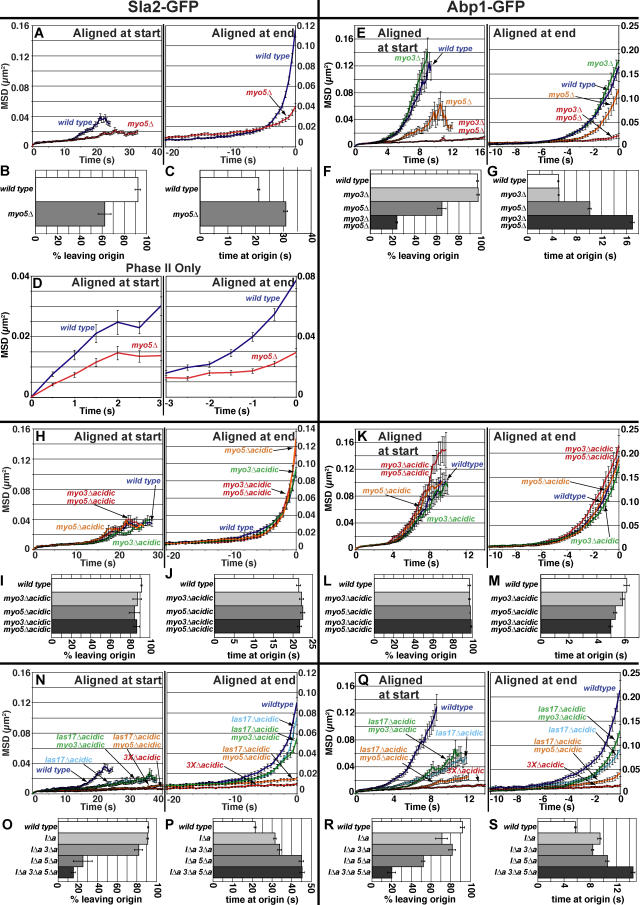
Quantitative Motion Analysis of Actin Patches in Type-I Myosin Mutants (A–G) Null mutants lacking one or both type-I myosin. Cells express Sla2-GFP (A–D) or Abp1-GFP (E–G). (A) MSD plots for wild-type and *myo5*Δ patches aligned at the start (left) or end (right) of their lifetimes. The curves on the left are truncated at the median lifetime. (B) Percentage of patches that leave the origin. Mean of values for three segregants is shown. (C) Average time at the origin, from the appearance of a patch until it moved away or disappeared. (D) Phase II movement only. For each patch, data prior to movement were removed. (E) MSD plots for wild-type and mutant patches aligned at the start (left) or end (right) of their lifetimes. On the left, plots are truncated at the median lifetime or 16 s, whichever was less. (F) Percentage of patches that leave the origin, as in (B). (G) Average time at the origin, as in (C). (H–M) Mutants lacking Arp2/3-binding regions of one or both type-I myosins, termed *myo3*Δ*acidic* and *myo5*Δ*acidic*. The panels are similar to those above, with cells expressing Sla2-GFP (H–J) or Abp1-GFP (K–M). (H and K) MSD plots for wild-type or mutant patches aligned at the start (left) or end (right) of their lifetimes. The curves on the left are truncated at the median lifetime. (I and L) Percentage of patches that leave the origin. Mean of values for three segregants is shown. (J and M) Average time at the origin, from the appearance of a patch until it moved away or disappeared. (N–S) Mutants lacking Arp2/3 binding regions of WASp/Las17 and type-I myosins. Cells express Sla2-GFP (N–P) or Abp1-GFP (Q–S). (N and Q) MSD plots for wild-type or mutant patches aligned at the start (left) or end (right) of their lifetimes. The curves on the left are truncated at the median lifetime. (O and P) Percentage of patches that leave the origin. Mean of values for three segregants is shown. (R and S) Average time at the origin, from the appearance of a patch until it moved away or disappeared. Strain numbers and number of patches: (A–C) Wild-type, YJC4787–9; 94, 79, and 90. *myo5*Δ, YJC4784–6; 103, 79, and 69. (D) Strains as in (A–C). Numbers of patches: wild-type – 88, 69, 85. *myo5*Δ – 75, 40, 36. (E–G) Wild-type, YJC4815–7; 121, 110, and 130. *myo3*Δ, YJC4409–11; 111, 115, and 105. *myo5*Δ, YJC4813–4; 120 and 99. *myo3*Δ *myo5*Δ, YJC4770–2; 143, 141, and 126. (H–J) Wild-type, YJC5566–8; 71, 107, and 83. *myo3*Δ*acidic*, YJC5563–5; 71, 75, and 101. *myo5*Δ*acidic*, YJC5554–6; 86, 71, and 62. *myo3*Δ*acidic myo5*Δ*acidic*, YJC5557–9; 83, 65, and 83. (K–M) Wild-type, YJC4848 and 50; 116 and 87. *myo3*Δ*acidic*, YJC4843–5; 119, 99, and 106. *myo5*Δ*acidic*, YJC4840–2; 102, 111, and 99. *myo3*Δ*acidic myo5*Δ*acidic*, YJC4837–9; 96, 93, and 100. (N–P) Wild-type, YJC5566–8; 71, 107, and 83. *las17*Δ*acidic*, YJC5551–3; 84, 64, and 69. *las17*Δ*acidic myo3*Δ*acidic*, YJC5560–2; 89, 71, and 68. *las17*Δ*acidic myo5*Δ*acidic*, YJC5548–50; 51, 66, and 57. *las17*Δ*acidic myo3*Δ*acidic myo5*Δ*acidic*, YJC5545–7; 43, 53, and 54. (Q–S) *las17*Δ*acidic*, YJC5208–10; 95, 76, and 89. *las17*Δ*acidic myo3*Δ*acidic*, YJC5438–40; 111, 104, and 109. *las17*Δ*acidic myo5*Δ*acidic*, YJC5441–3; 98, 80, and 84. *las17*Δ*acidic myo3*Δ*acidic myo5*Δ*acidic*, YJC5205–7; 71, 79, and 99.

The C termini of fungal type-I myosins, including Myo3 and Myo5 of budding yeast, have an acidic/DDW sequence that binds Arp2/3 complex (Figure 1A) [14,17,38,39]. These type-I myosins are capable of activating Arp2/3 in vitro when they are artificially connected to a WH2 domain or in the presence of the WH2-containing protein, Vrp1, the yeast WASp-interacting protein (WIP) [13,15]. To determine the role of type-I myosins in actin patch motility, we examined the effect of the deletion of the *myo3* and *myo5* genes on Abp1-GFP dynamics. *myo3*Δ cells were normal, by MSD analysis (Figure 3E), with no defects in the percentage of patches leaving the origin or the time that patches spent at the origin (Figure 3F–3G). In contrast, *myo5*Δ cells had substantial defects in Abp1-GFP motility (Figure 3E). The median lifetime of Abp1-GFP patches in the absence of Myo5 was found to be increased, and the distance of the movement of Abp1-GFP patches was decreased, in MSD plots with patch tracks aligned at the beginning and at the end of their lifetimes. The time that Abp1-GFP patches spent at the origin was increased in *myo5*Δ cells, and the percentage that moved away from the origin was decreased (Figure 3F–3G).

The effect of the loss of Myo5 on the early phases of actin patch motility was also examined using Sla2-GFP labeling. MSD plots of Sla2-GFP movement, aligned at the beginning and at the end of patch lifetimes, revealed decreased movement with an increase in median lifetime (Figure 3A). This defect was due in part to a decrease in the frequency with which Sla2-GFP patches left the origin and an increase in the time the patches spent at the membrane (Figure 3B–3C). To determine if the phase II movement of patches in *myo5*Δ cells was normal when they did leave the membrane, we isolated and analyzed movement of Sla2-GFP–labeled patches from the membrane. Phase II patch movement was found to be decreased in the *myo5*Δ cells by MSD analysis (Figure 3D).

Observations of cell growth and endocytosis have indicated that Myo3 and Myo5 have overlapping functions, with apparent redundancy in some cases [40,41]. To address this question, we examined Abp1-GFP patch behavior in *myo3*Δ *myo5*Δ double mutants. MSD curves showed a severe defect in patch motility, with a nearly complete loss of movement away from the origin and an increase in median patch lifetime, when compared to wild-type or single-mutant cells (Figure 3E). The percentage of Abp1-GFP patches leaving the membrane was very small, and the lifetime of patches at the origin was greatly increased (Figure 3F–3G). The number of patches leaving the membrane was not sufficient to permit a direct analysis of phase III movement. Taken together, these results show that although the function of Myo5 is distinct from that of Myo3, the proteins do have some functions in common. The results are consistent with the previous observation of endocytosis defects in *myo5*Δ but not *myo3*Δ single mutants, and with multiple observations that *myo5*Δ *myo3*Δ double mutants are more severely affected in growth and endocytosis than either single mutant alone [40,41].

### Arp2/3-Binding Regions of Type-I Myosin and WASp

To determine if the actin patch phenotypes of the type-I myosin null mutants, *myo3*Δ and *myo5*Δ, result from loss of Arp2/3 interaction, we truncated the acidic/DDW region of each type-I myosin. For Myo5, loss of the acidic/DDW region had no effect on phase I and II movement except for a small, statistically significant increase in movement in MSD plots of Sla2-GFP patch tracks aligned at the end of their lifetime (Figure 3H–3J). For phase III movement, which was assessed with Abp1-GFP, MSD curves showed a slight leftward shift when tracks were aligned at the start, but no change when aligned at the end (Figure 3K). Abp1-GFP patches remained at the origin for slightly less time in the mutant cells (Figure 3M; *p* = 0.0003), but the frequency with which patches moved away from the origin was normal (Figure 3L). These results were a surprising contrast to those for the *myo5*Δ null mutant, which had a strong defect in phase III and significant ones in phases I and II. For Myo3, a similar truncation of the acidic/DDW region had no effect on phase I or II of actin patch movement, which were examined with Sla2-GFP (Figure 3H–3J), or on phase III, which was examined using Abp1-GFP (Figure 3K–3M). These results were largely similar those for the *myo3*Δ null mutant and thus not surprising.

To determine if the absence of a phenotype in the single-mutant strains was a result of functional redundancy, we examined cells carrying both truncation mutations. Patch movement was again remarkably unaffected. The double mutant produced results very similar to those for the Myo5 single mutant for all three phases of patch life (Figure 3H–3J). During phase III, actin patches of double-mutant cells were slightly different from those of the Myo5 single mutant (Figure 3K–3M), but this difference barely achieved statistical significance.

Overall, the Arp2/3-binding regions of Myo3 and Myo5 were dispensable for function in this otherwise wild-type genetic background, in striking contrast to the effect of the complete loss of one or both proteins. Several lines of evidence suggest that the type-I myosins might function as a complex with WASp/Las17 and WIP/Vrp1, with overlapping function among the three Arp2/3-binding regions [14,17,42]. To test this possibility, we examined actin patch motility in haploid strains carrying a *las17*Δ*acidic* mutation in combination with *myo3*Δ*acidic* and/or *myo5*Δ*acidic* mutations.

First, we examined phases I and II with Sla2-GFP labeling. Patch movement in *las17*Δ*acidic myo3*Δ*acidic* cells was similar to that in *las17*Δ*acidic* cells during phases I and II (Figure 3N). Only small differences of marginal statistical significance were observed for the time that patches remained at the origin and for the frequency with which patches left the membrane (Figure 3P and 3O). In contrast, cells lacking the acidic/DDW regions of WASp/Las17 and Myo5 showed substantially greater defects than cells lacking only the acidic/DDW region of WASp/Las17. Patch movement in the double mutant was decreased, by MSD analysis, compared with that in the single mutant (Figure 3N). This defect resulted in large part from a decrease in the frequency with which patches left the origin to begin phase II movement (Figure 3O). In addition, patches in *las17*Δ*acidic myo5*Δ*acidic* cells spent more time at their point of origin before disappearing or moving away than did those of *las17*Δ*acidic* cells (Figure 3P).

In triple-mutant cells, which lack the acidic domains of WASp/Las17, Myo3, and Myo5, Sla2-GFP patch motility was decreased compared with double mutants (Figure 3N). The frequency with which triple-mutant patches left the origin was only slightly lower (Figure 3O), and the time that patches remained at the origin was similar (Figure 3P) when compared with *las17*Δ*acidic myo5*Δ*acidic* cells.

Phase III patch movement was examined in these mutants using Abp1-GFP. The loss of the acidic domain of Myo3 had little effect on the motility of Abp1-GFP patches in *las17*Δ*acidic* cells by MSD analysis (Figure 3Q). The frequency with which patches moved away from the origin and the time patches spent at the membrane were very similar (Figure 3R and 3S), similar to the results with Sla2-GFP labeling. If anything, loss of the acidic domain of Myo3 suppressed the phenotype of increased time that Abp1-GFP patches spent at the membrane in *las17*Δ*acidic* cells (Figure 3S, *p* = 0.0007).

In contrast, *las17*Δ*acidic myo5*Δ*acidic* double-mutant cells had greater defects in Abp1-GFP patch motility than did *las17*Δ*acidic* cells (Figure 3Q). The percentage of patches that moved away from their origin was decreased (Figure 3R; *p* = 0.04), and the time that patches spent at the membrane prior to moving away was increased (Figure 3S; *p* = 0.0004). Patches in *las17*Δ*acidic myo5*Δ*acidic* cells still retained a measurable level of dynamics and movement, so we asked if Myo3 was important in this context. Indeed, Abp1-GFP patch motility was decreased in *las17*Δ*acidic myo3*Δ*acidic myo5*Δ*acidic* triple-mutant cells compared to *las17*Δ*acidic myo5*Δ*acidic* cells, with a nearly complete loss of motility (Figure 3Q). The percentage of patches that moved away from the origin was less, and the time that patches remained at the origin was greater (Figure 3R–3S).

Together, the results indicate that the Arp2/3-binding regions of WASp and the type-I myosins do have a significant level of functional redundancy, consistent with the notion that they may act in a complex in which any and all of the three Arp2/3 complex interactions can be important. In previous studies suggesting the existence of such a complex, WIP, known as verprolin/Vrp1 in yeast, was also found in biochemical association. We found that a WIP null mutant, *vrp1*Δ, had essentially no patch movement, with Sla2-GFP or Abp1-GFP labeling (unpublished data), in agreement with another study [15]. Actin patches did still form in all of these mutants, including the WIP null mutant and the WASp/type-I myosin triple mutant, showing that actin filaments can still polymerize but not with the dynamic control needed to achieve movement.

### A Role for Pan1 in the Early Phases of Patch Assembly

**Figure 4:**
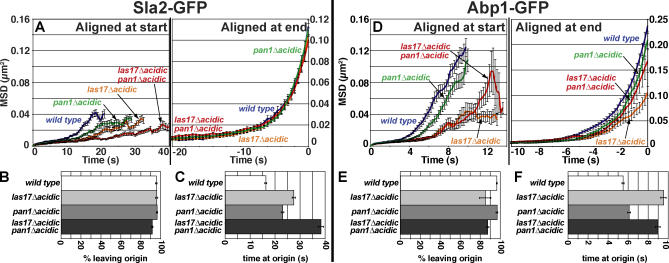
Quantitative Analysis of Actin Patch Motility in Cells Lacking the Arp2/3-Binding Regions of Pan1 and WASp/Las17 Cells express Sla2-GFP (A–C) or Abp1-GFP (D–F). **(**A and D) MSD plots for wild-type and mutant patches aligned at the start (left) or end (right) of their lifetimes. The curves on the left are truncated at the median lifetime. (B and E) Percentage of patches that leave the origin. Mean of values for three segregants is shown. (C and F) Average time at the origin, from the appearance of a patch until it moved away or disappeared. Strain numbers and numbers of patches: (A–C) Wild-type, YJC5707 - 9; 66, 84, and 69. *las17*Δ*acidic*, YJC5713–5; 100, 95, and 104. *pan1*Δ*acidic*, YJC5710–2; 103, 76, and 74. *las17*Δ*acidic pan1*Δ*acidic*, YJC5716–8; 100, 86, and 91. (D–F) Wild-type, YJC5719–22; 99, 100, 114, and 98. *las17*Δ*acidic*, YJC5726, 8 & 9; 95, 88, and 84. *pan1*Δ*acidic*, YJC5723–5; 102, 94, and 139. *las17*Δ*acidic pan1*Δ*acidic*, YJC5730–3; 77, 70, 50, and 50. Error bars are ± standard error.

The endocytic adaptor protein Pan1 has an acidic/DDW region for binding the Arp2/3 complex, along with two EH domains, a coiled-coil region, and a WH2 domain [43]. Pan1 is essential for viability in yeast, and because the loss of endocytosis does not appear to be lethal, this suggests that Pan1 may have other functions. To investigate the possible importance of Pan1′s interaction with the Arp2/3 complex in actin patch dynamics, the acidic/DDW region of the Pan1 protein was removed (Figure 1A). The *PAN1* gene was mutated at its endogenous locus in a diploid strain and tetrad dissection produced haploid mutant segregants that grew well (Protocol S1). In haploid mutant *pan1*Δ*acidic* cells labeled with Sla2-GFP, the time that patches remained at their origin was slightly increased (Figure 4C), and MSD plots of tracks aligned at the start of their lifetimes showed decreased movement (Figure 4A). The percentage of patches leaving the origin was normal (Figure 4B), as was MSD analysis with curves aligned at the end of their lifetimes (Figure 4A). Examining only the data for movement away from the membrane in phase II, we found that patch movement was also normal in the mutant (unpublished data). Thus, only the earliest stages of patch dynamics were affected by removing the Arp2/3 binding region of Pan1.

WASp / Las17 was also important in the early stages of the actin patch life cycle, as described above, so we combined the *pan1*Δ*acidic* and *las17*Δ*acidic* mutations. Double-mutant haploid cells had a more severe defect than did either single mutant, in terms of the time that patches spent at their origin (Figure 4C) and MSD analysis with curves aligned at the start (Figure 4A, left). The percentage of patches leaving the origin was decreased slightly (Figure 4B), and MSD analysis with curves aligned at the right was normal, similar to the single mutants (Figure 4A, right). This enhanced phenotype in the double mutant is consistent with synthetic interactions between *pan1* and *las17* mutations in terms of cell growth [42].

With Abp1-GFP labeling to examine phase III behavior, *pan1*Δ*acidic* single-mutant cells displayed a defect in MSD plots aligned at the left, no defect in MSD plots aligned at the right (Figure 4D), a decrease in the percentage of patches that moved away from the origin (Figure 4E), and a slight increase in the time that patches remained at the origin before moving away or disappearing (Figure 4F; *p* = 0.0005). These results are consistent with the phase I and II results for this mutant, based on the Sla2-GFP labeling result above, and they indicate that phase III patch behavior was normal in the *pan1*Δ*acidic* single mutant. For double-mutant *las17*Δ*acidic pan1*Δ*acidic* cells labeled with Abp1-GFP, the early phases of actin patch dynamics were similar to those of *las17*Δ*acidic* cells, based on the early time points in MSD plots aligned at the left. The time that patches spent at the membrane and in the percentage of patches that moved away from the origin was also similar (Figure 4E and 4F). However, double-mutant cells showed an increase in phase III movement, compared to *las17*Δ*acidic* cells, at later time points in MSD plots, aligned at the start or end of patch life (Figure 4D). This rescue is the result of a combination of an increase in the percentage of patches making movements beyond 200 nm and an increase in the movement during phase III (unpublished data). Thus, in this respect, the *pan1*Δ*acidic* mutation suppressed the phenotype of the *las17*Δ*acidic* mutation, suggesting that Pan1 and Las17 have opposing actions on Arp2/3 complex in this later phase of patch lifetime.

### Abp1 Has a Role in the Early Phases of Patch Dynamics

Abp1 is present at the patch just before movement of the patch away from the membrane to the end of its life. Abp1 has two acidic/DDW regions that bind the Arp2/3 complex (Figure 1A) and an ADFH domain that binds F-actin [44]. In early studies, *abp1*Δ null mutants were nearly normal in many respects, but the *abp1*Δ mutation has been found to have genetic interactions with *sla1*Δ, *sla2*, and *las17*Δ mutations [45]. Actin patches of *abp1*Δ mutants, labeled with Sla1-GFP, showed an increase in the distance that Sla1-GFP moved from the membrane and an increase in Sla1 and Sac6 lifetimes [23].

**Figure 5:**
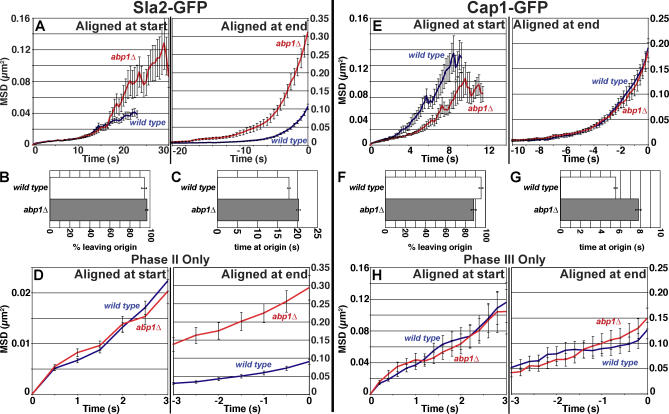
Quantitative Motion Analysis of Actin Patches in Null Mutants Lacking Abp1 Cells express Sla2-GFP (A–D) or Cap1-GFP (E–H). (A and E) MSD plots for wild-type and mutant patches aligned at the start (left) or end (right) of their lifetimes. The curves on the left are truncated at the median lifetime. (B and F) Percentage of patches that leave the origin. Mean of values for three segregants is shown. (C and G) Average time at the origin, from the appearance of a patch until it moved away or disappeared. (D) Phase II movement only. For each patch, data prior to movement were removed. Only patches that moved away from the origin were included. (H) Phase III movement only. For each patch, data prior to the time the patch traveled 200 nm were removed. Only patches that moved more than 200 nm from the origin were included. Strain numbers and number of patches: (A–C) Wild-type, YJC5457–9; 157, 141, and 151. *abp1*Δ, YJC5454–6; 95, 118, and 85. (D) Strains as in (A–C). Numbers of patches: wild-type – 153, 135, 135. *abp1*Δ – 92, 112, 84. (E–G) Wild-type, YJC5681–3; 74, 75, and 72. *abp1*Δ, YJC5684–6; 62, 58, and 57. (H) Strains as in (E–G). Numbers of patches: wild-type – 64, 69, 64. *abp1*Δ – 44, 43, 51 Error bars are ± standard error.

We found that Sla2-GFP patches of *abp1*Δ cells remained longer at their origin (Figure 5C; *p* = 0.0002), and the percentage of patches that left their origin was normal (Figure 5B). By isolating the data for patch movement away from the membrane in phase II, we found that initial movement of patches away from the membrane was normal (Figure 5D, left) and that, remarkably, the final extent of movement was greatly increased (Figure 5D, right). Consistent with these observations, MSD analysis of the complete tracks showed an increase in the median lifetime of the patches and greater movement away from the origin (Figure 5A). One explanation for the increase in median lifetime and movement is that Sla2-GFP remains on the patch for a longer time in the absence of Abp1.

Phase III of actin patch movement in *abp1*Δ cells was examined using Cap1-GFP as a marker; the behavior of Cap1-GFP patches essentially mirrors that of Abp1-GFP patches in wild-type cells [23,33]. The time that patches spent at their origin was increased (Figure 5G), and the percentage of patches moving away from the membrane was normal (Figure 5F, *p* = 0.08), as seen with Sla2-GFP labeling above. MSD plots of the complete lifetime were decreased in *abp1*Δ cells, for curves aligned at the start of their lifetime, but not at the end (Figure 5E). We isolated the data for patch movement beyond 200 nm, and the results for *abp1*Δ cells were identical to those for wild-type cells (Figure 5H). The Cap1-GFP results indicate a delay in the initiation of patch movement with normal movement after leaving the membrane for the *abp1*Δ mutant.

Considering all the data, for Sla2-GFP and Cap1-GFP labeled patches, Abp1 appears to be important to promote patch assembly before leaving the membrane, but not for patch movement after leaving the membrane. In addition, Abp1 appears to play a role in the removal of Sla1 and Sla2 from the patch after the initial movement into the cytoplasm.

### Coronin as an Inhibitor of Actin Assembly

**Figure 6:**
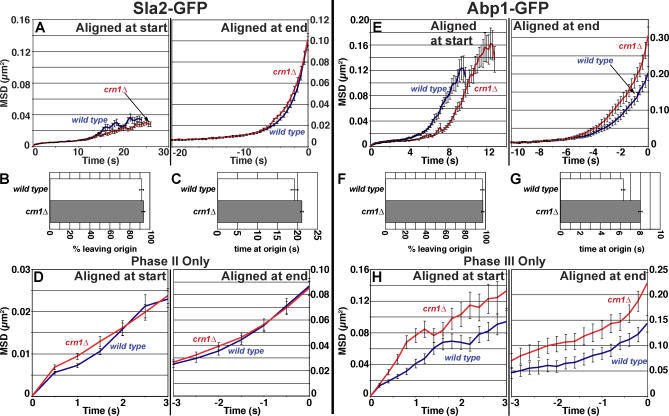
Quantitative Motion Analysis of Actin Patches in Null Mutants Lacking Coronin, Crn1 Cells express Sla2-GFP (A–D) or Abp1-GFP (E–H). (A and E) MSD plots for wild-type and mutant patches aligned at the start (left) or end (right) of their lifetimes. The curves on the left are truncated at the median lifetime. (B and F) Percentage of patches that leave the origin. Mean of values for three segregants. (C and G) Average time at the origin, from the appearance of a patch until it moved away or disappeared. (D) Phase II movement only. For each patch, data prior to movement were removed. Only patches that moved away from the origin were included. (H) Phase III movement only. For each patch, data prior to the time the patch traveled 200 nm were removed. Only patches that moved more than 200 nm from the origin were included. Strain numbers and number of patches: (A–C) Wild-type, YJC5449 - 51; 177, 192, 173. *crn1*Δ, YJC5446–8; 155, 202, 147. (D) Strains as in (A–C). Numbers of patches: wild-type – 165, 182, 152. *crn1*Δ – 143, 184, 142. (E–G) Wild-type, YJC4297–9; 74, 62, 57. *crn1*Δ, YJC4235, 6 & 9; 88, 74, 72. (H) Strains as in (E–G). Numbers of patches: wild-type – 67, 59, 49. *crn1*Δ – 84, 68, 69 Error bars are ± standard error.

Coronin/Crn1 has been shown in vitro to inhibit the activity of the Arp2/3 complex for nucleation of actin polymerization [21]. To determine if coronin has such a role in actin patch dynamics, we examined *crn1*Δ null mutant cells. Phases I and II of patch motility, examined with Sla2-GFP, were nearly normal in most respects, with only a slight decrease in the MSD plot, a slight increase in median lifetime (Figure 6A, left), and a slight increase in time at the origin (Figure 6C; *p* = 0.001). With Abp1-GFP labeling of patches, the time spent at the origin was increased but not the probability of leaving the origin (Figure 6F and 6G), similar to the results with Sla2-GFP. Together, these results suggest a minor delay in the transition from phase I to II when coronin is absent, suggesting that coronin promotes the ability of patches to move off the membrane.

Phase III dynamics in *crn1*Δ mutant cells, examined using Abp1-GFP, showed a rightward shift in the MSD plot with curves aligned at the start for the mutant (Figure 6E, left). The plot extended to greater distances and longer times, suggesting that patches moved farther and longer in the absence of coronin. In support of this idea, when the curves were aligned at the end of patch lifetime, the MSD plot showed increased movement of *crn1*Δ patches (Figure 6E, right). Isolating the data for movement of Abp1-GFP patches in phase III, *crn1*Δ patches showed more movement by MSD analysis, with curves aligned at the start or end of their lifetime (Figure 6H). Thus, coronin appears to inhibit the movement of patches after they move away from the membrane and into the cytoplasm.

## Discussion

**Figure 7:**
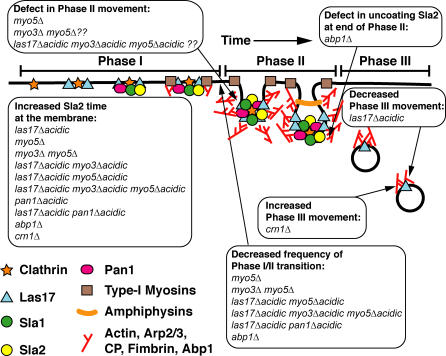
A Model for Endocytosis and Actin Patch Assembly, with a Summary of the Requirements for Individual Arp2/3 Regulatory Proteins During phase I, almost all of the Arp2/3 regulators have a role in promoting assembly. At this stage, Arp2/3 regulators presumably act to establish a proper actin network with appropriate filament lengths and branch density. During phase II, Arp2/3 regulation is complex. Mutation of no single Arp2/3 regulator affected this stage, but simultaneous mutation of the Arp2/3 binding regions of WASp/Las17, Myo3, and Myo5 did disrupt this movement. Capping protein also has a role at this stage [33]. During phase III, several lines of evidence support a model where actin filaments, nucleated at the actin patch, drive movement of the vesicle through the cytoplasm. In this model, barbed ends are oriented toward the vesicle (see Discussion).

Cells have a number of proteins with the biochemical ability to regulate the Arp2/3 complex. Whether these proteins have such roles in cells and, if so, how those roles are manifested and coordinated are important questions in understanding the cell biology of actin assembly and motility. Our results demonstrate that the collection of Arp2/3 regulatory proteins in yeast have largely distinct functions in generating actin assembly to create force and movement during endocytosis. In some situations, individual functions can overlap, increasing the robustness of the system. Figure 7 summarizes the results, illustrating the roles of specific regulators in the stereotyped series of processes that compose the assembly and movement of cortical actin patches as part of endocytosis.

### Assembly of the Actin Patch

Early in the life of an actin patch, a series of proteins are recruited to a location on the plasma membrane, assembling the machinery required for endocytosis [24]. During this time, the actin patch appears to be tethered or corralled, undergoing random movements confined to a small area [24,33]. Assembly of a dendritic network of actin filaments proceeds [24,33], which appears to be needed for the movement of the actin patch and endocytic vesicle away from the membrane.

Our results show that, on some level, essentially all of the Arp2/3 regulatory proteins play a role in these initial phases of the process. Mutations in these proteins alter the amount of time that patches persist at the site of their appearance, undergoing tethered movement, which is termed phase I. For the WASp Las17 and the EH domain protein Pan1, mutations of the Arp2/3-binding region prolonged the duration of phase I, as did null mutations for the type-I myosin Myo5, coronin, and Abp1. Mutations in the Arp2/3-binding motif of Las17 delays the appearance of Abp1-GFP at patches, suggesting that although the other Arp2/3 activators can eventually assemble an actin filament network, they do so less efficiently without Las17.

Combinations of Arp2/3-binding mutations reveal the potential for overlapping function among the regulators. For example, combining the *las17*Δ*acidic* mutation with the *pan1*Δ*acidic*, the *myo5*Δ*acidic*, or the *myo5*Δ*acidic myo3*Δ*acidic* mutations further prolonged the time for patch assembly in phase I. Arp2/3 regulators are likely required at this stage to ensue the proper targeting and activity level of the Arp2/3 complex to generate a functional actin network for movement. In their absence, the network may not have the proper branch density and filament length required to initiate invagination and/or movement.

### Patch Movement away from the Plasma Membrane: WASp and Type-I Myosin

The first nonrandom movement of an actin patch is a short one away from the plasma membrane into the cytoplasm, which has been hypothesized to represent membrane invagination to form an endocytic cup [24]. Complete deletions of the type-I myosin genes had the greatest effect on this movement. Loss of Myo5 decreased the probability that a patch would undergo phase II movement, and it lessened the extent of movement per se. Loss of both Myo3 and Myo5 resulted in almost no movement away from the cortex, supporting a model that type-I myosins are needed to power this movement prior to vesicle scission [15].

Given the severity of the phenotypes in the type-I myosin null mutants, we were surprised to find that truncation of the type-I myosin Arp2/3-binding regions had little or no effect. Myo3 and Myo5 have been suggested to function in coordination with or in parallel with WASp/Las17, perhaps as a multisubunit complex [14,17,42]. When we combined Arp2/3-binding mutations of WASp/Las17 with those of type-I myosins, the phenotype was enhanced, consistent with that view. In *las17*Δ*acidic myo5*Δ*acidic* and *las17*Δ*acidic myo3*Δ*acidic myo5*Δ*acidic* cells, actin patches failed to move away from the origin. Verprolin/Vrp1, the yeast homologue of WIP was suggested to function as a scaffold or central component of such a multisubunit complex [14]. In support of that view, loss of Vrp1 caused a complete loss of actin patch movement [15]. In our hands, the loss of Vrp1 caused an almost complete loss of inward movement in plots of MSD (unpublished data).

Many Arp2/3 regulators are dispensable for phase II movement. Deletion of the acidic domain of Las17 or Pan1, independently or together, had no effect. The loss of neither coronin nor Abp1 affected this movement. Loss of Abp1 did result in Sla2-GFP–labeled patches moving farther away from the cortex than normal, which supports the view that Abp1 may help to remove early components such as Sla1 and Sla2 from the patch [23].

Phase II may not depend on precise regulation of Arp2/3 localization or activation, because no single Arp2/3 binding mutant had a detrimental effect on this movement. However, previous studies of the capping protein suggest that the regulation of the number of free barbed ends is critical for normal movement during phase II. Furthermore, in the absence of the Arp2/3-binding regions of Las17/WASp and the type-I myosins, phase II movement fails to occur. This failure in phase II may reflect a requirement for Arp2/3 activation during phase II movement itself or it may also reflect a failure to establish a proper network during the phase I. In addition, the motor activities of the type-I myosins appear to play some role in the in initial movement of patches away from the membrane [15].

### Patch Movement in the Cytoplasm

After the actin patch departs the plasma membrane, it moves faster and farther, through the cytoplasm. When the Arp2/3-binding region of WASp/Las17 was truncated, actin patches had a severe defect in phase III movement, which is important in light of the relative normalcy of phase II movement for this mutant. The simplest interpretation of these results is that WASp/Las17 remains bound to the endocytic vesicle as it moves through the cytoplasm, analogous to the situation for the movement of pathogens in cells or beads in cell extracts.

GFP-WASp fusions at the N and C termini were not seen to leave the membrane in this manner in previous studies in budding and fission yeast, including some in our lab [24,33,35]. We observed that tagging WASP/Las17 at either end with GFP results in defects in actin patch motility reminiscent of those seen in *las17*Δ*acidic* strains (Figures S2–S4). We also observed that overexpressed Las17, tagged at the N terminus, did leave the membrane and move about the cytoplasm, in the manner of actin patches and endocytic vesicles (Figure 2I–2J). To be fair, the fluorescence of these foci of GFP-Las17 was weak, and Abp1-tdimer2 patch movement in these cells was defective. Therefore, it remains a possibility that Las17 may not normally leave the membrane with patches.

Our results with coronin address this question of how the patch moves through the cytoplasm. The coronin null mutant showed increased patch movement during phase III, which is consistent with the prediction from biochemical studies that coronin inhibits the activity of Arp2/3 complex. Yeast Arp2/3 complex is highly active in the absence of any activator when actin from yeast is used [11], so an inhibitor, namely coronin, may be quite important in this system. The loss of the capping protein resulted in a specific defect of phase III movement [33], which is also consistent with the model. Together, these results with WASp/Las17, coronin, and capping protein provide evidence that Arp2/3-mediated actin assembly powers the movements of phase III, which supports the relevance of the dendritic nucleation model for this process. Several key components of the dendritic nucleation model are known to be present on patches during phase III movement, including actin, the Arp2/3 complex, capping protein, and now WASp/Las17 [23,24,30,33].

The location of actin filament nucleation and the orientation of the actin filaments during actin patch assembly and movement is an important question. When endocytosis is blocked in *sla2*Δ cells, actin “comet tails” appear at the plasma membrane, with growing barbed ends oriented toward the membrane [24]. However, it is unclear where actin filaments are nucleated when endocytosis is proceeding normally, especially during invagination and during movement after the patch leaves the plasma membrane. No actin connection between the plasma membrane and a phase II or III patch can be seen. If filaments growing at the plasma membrane were responsible for this movement, the newest parts of the network would be at the membrane, and would likely be relatively bright when monitored by fluorescent fusion proteins. This is not the case. Previous results show that actin cables do not drive patch movement in the cytoplasm [33]. We therefore favor a model where actin filaments are being nucleated at the surface of the endocytic vesicle, during and after invagination*.*

We observed some longer-lived GFP-Las17–labeled particles moving in the interior of the cell without first seeing them leave the plasma membrane. For actin patches, this type of observation was very rare. Whether these GFP-Las17 particles represent a later stage of actin patches, endocytic vesicles, or another cellular compartment remains to be determined. The character of the movement of these particles is reminiscent of what has been seen by studying the membrane receptor Ste2 or the lipid dye FM4–64, which follow endocytic trafficking pathways [33,36]. In previous studies, the movement of Ste2-GFP particles was impaired when WASp/Las17 was truncated or when Lsb6, a Las17-binding protein, was absent [36,37], which also supports the hypothesis that Las17 is needed to drive Ste2 vesicles.

## Materials and Methods

### Yeast strains.

The strains used in this study are listed in Table S1 and their construction is described in Protocol S1.

### Microscopy.

For each genotype, two or three mutant and wild-type haploid segregants from a heterozygous diploid were tested. For each segregant, movies were collected from eight different cells. Strains were grown overnight in YPD at 25 °C or 30 °C to an optical density at 600 nm (OD_600_) of 0.1–0.5. Cells were harvested by centrifugation at 82*g*, suspended in SD-complete media, placed on 2% agarose pads made with SD-complete, and covered with a number 1 coverslip as described [33]. GFP fluorescence movies were made using a spinning-disc confocal microscope system consisting of an upright microscope (BX52, Olympus) with a PlanApo100X 1.4 NA oil immersion objective, a CSU10 Yokogawa spinning disc head (Solomere Technology), and an intensified charge coupled device (CCD) video camera (XR Mega10 S30 camera, Stanford Photonics). Two color images were collected sequentially using a LMM5 Laser Merge Module (Spectral Applied Research), a multipass dichroic mirror, and emission filters in a Lambda 10–3 high-speed filter wheel (Sutter Instruments). The temperature was maintained at 30 °C. Large budded cells were selected for observation. Images were collected from a single focal plane at the equator of the cell. Abp1-GFP, Abp1-tdimer2, Sla2-GFP, and GFP-Las17 movies were collected at frame rates of 5/s, 5/s, 2/s, and 2/s, respectively. For quantitative motion analysis, 200 consecutive frames were collected. Images were collected using QED In Vivo software (Media Cybernetics), except for the experiments in Figure 4D–4F and Figure 2I–2K, which were collected using Piper Imaging.

### Computer-assisted patch tracking and quantitative motion analysis.

GFP-labeled actin patches in movies were tracked using previously described software [31]. The experimenter verified every patch track using an ImageJ (National Institutes of Health) plug-in that overlaid the position information from the tracking software onto the original movie, and necessary corrections were made by hand. The data were then imported into an Excel spreadsheet (Microsoft). Tracks were retained for analysis if they met the following criteria: (1) The patch was observed for at least 30 frames (6 s for Abp1-GFP and 15 s for Sla2-GFP). (2) The patch originated near the cortex. (3) The patch disappeared during the movie. (4) The patch was readily distinguished from other patches. These criteria enrich our sample for patches whose entire lifetime is captured in the movie. Zero time for a track was defined as the time at which the patch was first observed, and the end point was defined as the last time point at which the patch was observed.

The character and extent of the motion of GFP-labeled patches was analyzed first with plots of MSD versus time. To calculate MSD, the square of the distance of each patch from its origin was calculated at each time point. The squared displacement values were averaged for all the patches from all the cells of each segregant of a particular genotype. The degree of variation among individual tracks was high, as seen in previous studies, so we averaged the data for 150 to 542 patches for each genotype. To test for statistical significance, MSD curves from each segregant of one genotype were first compared by analysis of variance (ANOVA) to ensure that they yielded the same result. A grand MSD versus time plot was then generated, averaging the data for all of the patches from all of the segregants of a given genotype within one experiment. Before averaging, the squared displacement values for each patch were aligned at the beginning or end of the track lifetime. Examples of how a set of data was aligned in the two ways before averaging are shown in Figure 1C. Plots aligned “at the start” or “on the left” provide information about the behavior of patches in the early part of their life. These curves are truncated at the time when 50% of the patches have disappeared, representing the median lifetime. Plots aligned “at the end” or “on the right” allow for a better understanding of the motion of GFP-labeled actin patches later in their lifetime, away from the origin. Comparisons between genotypes were evaluated using Student's *t*-test.

The patch tracking data were analyzed in additional ways. For individual patch track data of X-Y position and time, we determined if a patch left its origin and how long it remained at its origin before it moved away or disappeared. To analyze “phase II only” data after initiation of movement, the data for Sla2-GFP patches after they moved away from the origin were selected. MSD versus time plots were generated with these data. To analyze “phase III only,” we selected the data for Abp1-GFP labeled patches after they moved more than 200 nm away from the origin. MSD plots were generated.

## Supporting Information

Figure S1 Histogram of the Time between Sla2-GFP and Abp1-tdimer2 Recruitment to Actin Patches All strains express Sla2-GFP and Abp1-tdimer2. The median time for Abp1-tdimer2 recruitment +/- presented. Strain numbers and the number of patches were as follows: wild-type, YJC6044, 6046; 212 and 126, respectively. *las17*Δ*acidic*, YJC6041, 6043; 132 and 131 respectively.
(536 KB AI).

Figure S2 Quantitative Motion Analysis of Abp1-tdimer2 Patches in Wild-Type and *las17-GFP* Cells All cells express Abp1-tdimer2, and the movement of Abp1-tdimer2 was analyzed. Experiments were done using a different fluorophore to mark Abp1, and data were collected on a different microscope, so MSD values do not match those in other figures. (A) MSD plots for patches aligned at the start (left) or end (right) of their lifetimes. The curves on the left are truncated at the median lifetime.
(B) Percentage of patches that leave the origin. Mean of values for three segregants is shown.
(C) Average time at the origin, from the appearance of a patch until it moved away or disappeared.
(D) Phase III movement only. For each patch, data prior to the time the patch traveled 200 nm were removed. Only patches that moved at least 200 nm are included. Strain numbers and the number of patches were as follows: wild-type, YJC5950–2; 59, 79, and 69, respectively. *las17-GFP*, YJC5953–5; 87, 83, and 85. (D) Strains as in (A–C). Numbers of patches: wild-type – 57, 75, 66. *las17-GFP* – 81, 69, 76. Error bars are ± standard error.
(412 KB AI).

Figure S3 Quantitative Motion Analysis of Abp1-tdimer2 Patches in Wild-Type and *GFP-las17* Cells GFP-Las17 is expressed under the control of the endogenous promoter at the locus. All cells express Abp1-tdimer2, and the movement of Abp1-tdimer2 was analyzed.
(A) MSD plots for patches aligned at the start (left) or end (right) of their lifetimes. The curves on the left are truncated at the median lifetime.
(B) Percentage of patches that leave the origin. Mean of values for three segregants is shown.
(C) Average time at the origin, from the appearance of a patch until it moved away or disappeared.
(D) Phase III movement only. For each patch, data prior to the time the patch traveled 200 nm were removed. Only patches that moved at least 200 nm are included. Strain numbers and the number of patches were as follows: wild-type, YJC6180–2; 112, 104, and 110, respectively. *GFP-las17*, YJC6183–5; 126, 108, and 92. (D) Strains as in (A–C). Numbers of patches: wild-type – 106, 95, 104. *las17-GFP* – 116, 90, 77. Error bars are ± standard error.
(425 KB AI).

Figure S4 Quantitative Motion Analysis of Abp1-tdimer2 Patches in Wild-Type and *GAL1-GFP-las17* Cells All cells were grown in the presence of galactose as the sole carbon source and express Abp1-tdimer2. The movement of Abp1-tdimer2 was analyzed.
(A) MSD plots for patches aligned at the start (left) or end (right) of their lifetimes. The curves on the left are truncated at the median lifetime.
(B) Percentage of patches that leave the origin. Mean of values for three segregants is shown.
(C) Average time at the origin, from the appearance of a patch until it moved away or disappeared.
(D) Phase III movement only. For each patch, data prior to the time the patch traveled 200 nm were removed. Only patches that moved at least 200 nm are included. Strain numbers and the number of patches were as follows: wild-type, YJC6023–4; 37, 70, and 47, respectively. *GAL1-GFP-las17*, YJC6021–2; 66 and 66. (D) Strains as in (A–C). Numbers of patches: wild-type – 29, 65, 41. *GAL1-GFP-las17* – 50, 46. Error bars are ± standard error.
(419 KB AI).

Protocol S1 Description of the Generation of Strains Used (55 KB DOC)

Table S1 Strains Used in This Study (281 KB DOC)

Video S1 Sla2-GFP in Wild-Type Strain YJC5457 10X speed. Video speed 20 frames/s. Data collected at 2 frames/s.
(9.8 MB MOV)

Video S2 Abp1-GFP in Wild-Type Strain YJC4816 4X speed. Video speed 20 frames/s. Data collected at 5 frames/s.
(9.1 MB MOV)

Video S3 GFP-Las17 in Strain YJC5736 Includes examples of GFP-Las17, expressed under control of the GAL1 promoter, moving away from the membrane. 10X speed. Video speed 20 frames/s. Data collected at 2 frames/s.
(3.3 MB MOV)

Video S4 GFP-Las17 in Strain YJC5737 Includes examples of GFP-Las17, expressed under control of the GAL1 promoter, moving away from the membrane. 10X speed. Video speed 20 frames/s. Data collected at 2 frames/s.
(7.3 MB MOV)

Video S5 GFP-Las17 (Green) and Abp1-tdimer2 in Strain YJC6179 Includes an example of GFP-Las17, expressed under control of the GAL1 promoter, moving off the membrane with Abp1-tdimer2 (arrowhead). 2.5X speed. Video speed 5 frames/s. Data collected at 1 frame every 2 s. Red and green channels were collected sequentially at 1-s intervals.
(310 KB MOV)

Video S6 GFP-Las17 in Strain YJC5737, Expressed under Control of the GAL1 Promoter Includes examples of long-lived particles moving in the cytoplasm that do not originate at the membrane (arrowheads). 10X speed. Video speed 20 frames/s. Data collected at 2 frames/s.
(3.5 MB MOV)

## Abbreviations

GFP: green fluorescent protein
MSD: mean square displacement
WIP: WASp-interacting protein
