# Supplementary material for: Distinct Roles for Arp2/3 Regulators in Actin Assembly and Endocytosis
Source: PLoS Biol. 2008 Jan 3;6(1):e1. doi: 10.1371/journal.pbio.0060001 (PMC2156081; doi:10.1371/journal.pbio.0060001)
Supplement: Supplementary file 1 [file pbio.0060001.sg001.pdf]

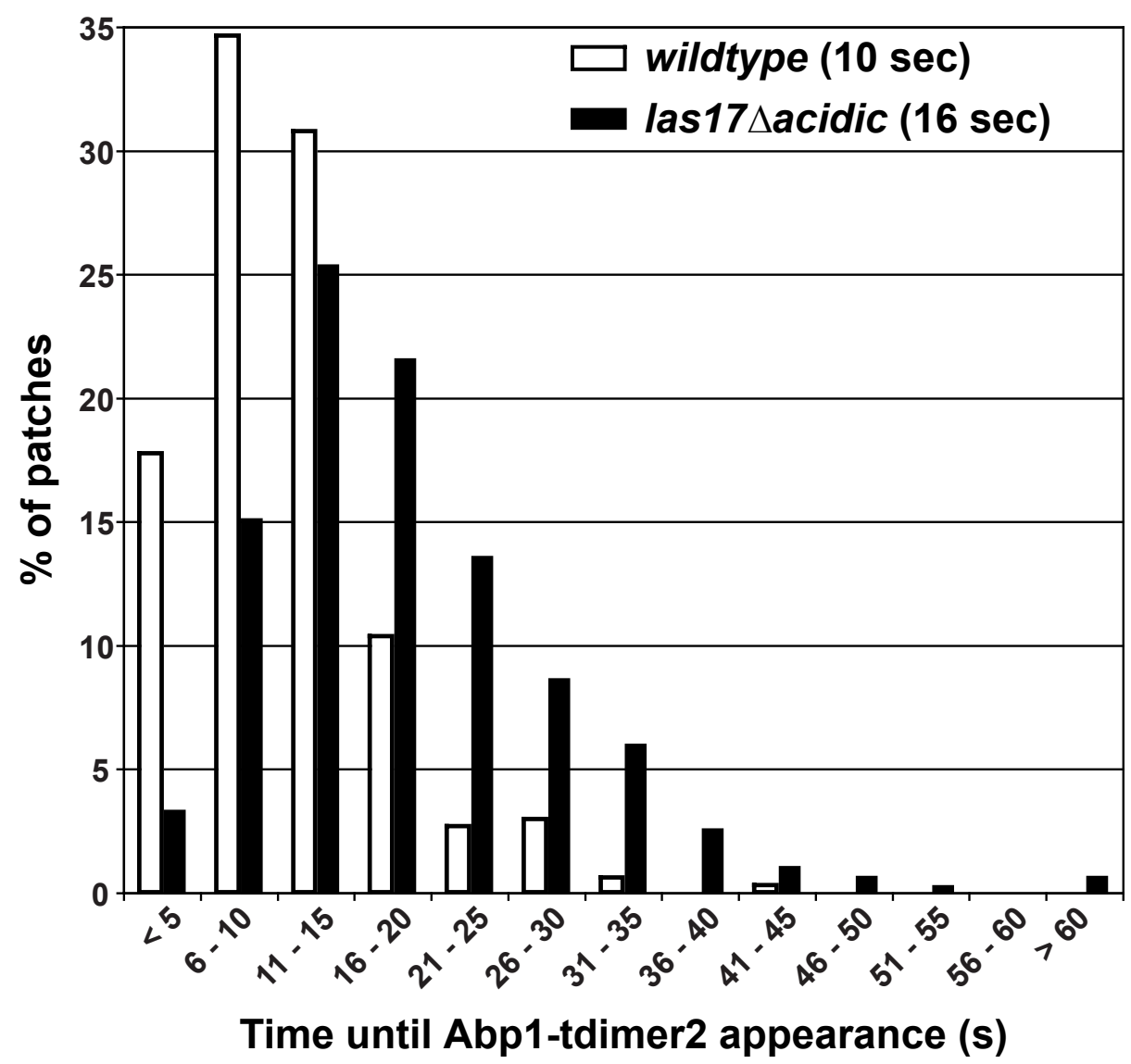
wildtype (10 sec)
las17Δacidic (16 sec)
% of patches
35
30
25
20
15
10
5
0
< 5
6 - 10
11 - 15
16 - 20
21 - 25
26 - 30
31 - 35
36 - 40
41 - 45
46 - 50
51 - 55
56 - 60
> 60
Time until Abp1-tdimer2 appearance (s)
