# Supplementary material for: Distinct Roles for Arp2/3 Regulators in Actin Assembly and Endocytosis
Source: PLoS Biol. 2008 Jan 3;6(1):e1. doi: 10.1371/journal.pbio.0060001 (PMC2156081; doi:10.1371/journal.pbio.0060001)
Supplement: Supplementary file 2 [file pbio.0060001.sg002.pdf]

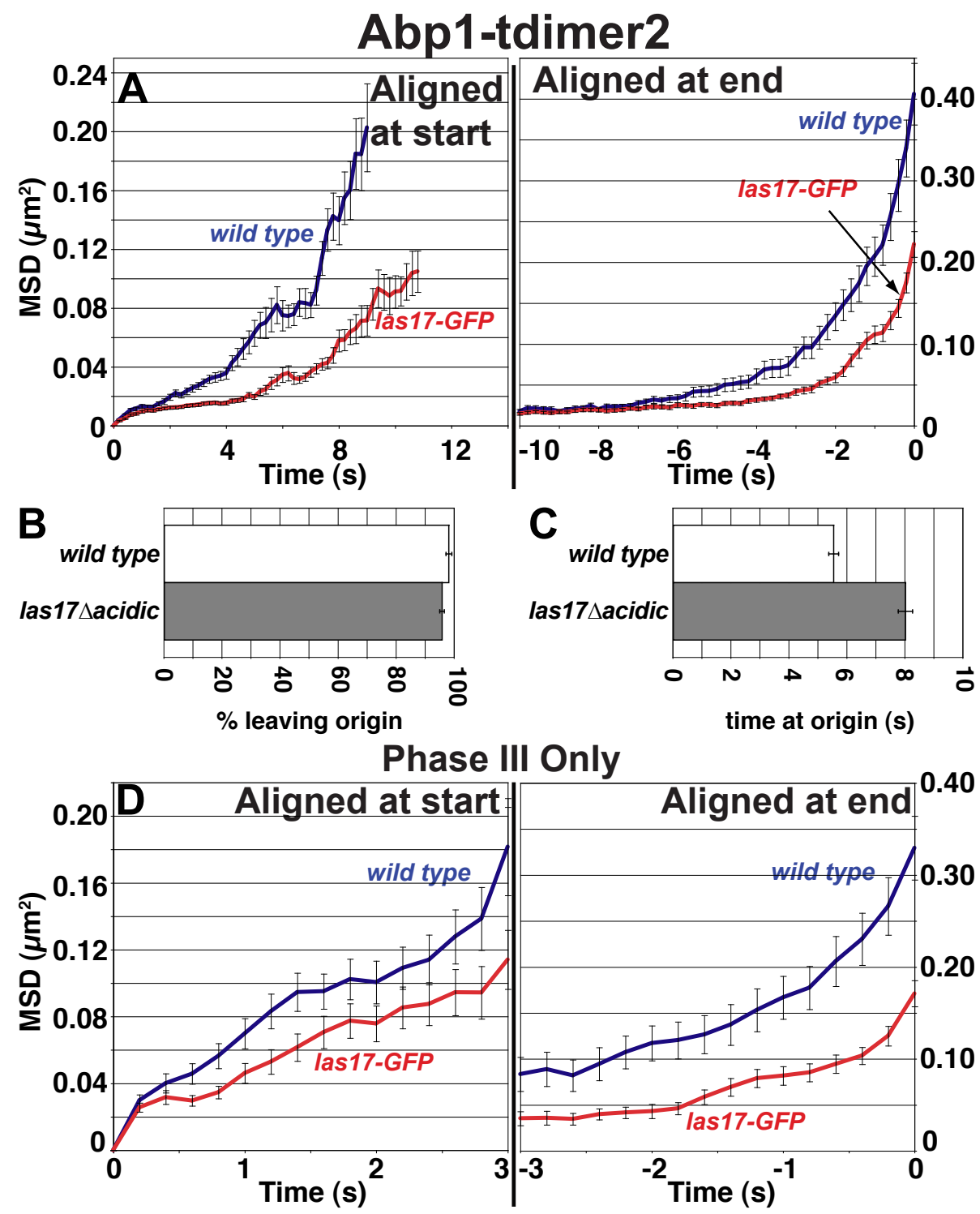
Abp1-tdimer2
A
Aligned at start
wild type
las17-GFP
MSD (μm²)
Time (s)
Aligned at end
B
wild type
las17Δacidic
% leaving origin
C
time at origin (s)
Phase III Only
D
Aligned at start
Aligned at end
