# Supplementary material for: Distinct Roles for Arp2/3 Regulators in Actin Assembly and Endocytosis
Source: PLoS Biol. 2008 Jan 3;6(1):e1. doi: 10.1371/journal.pbio.0060001 (PMC2156081; doi:10.1371/journal.pbio.0060001)
Supplement: Supplementary file 3 [file pbio.0060001.sg003.pdf]

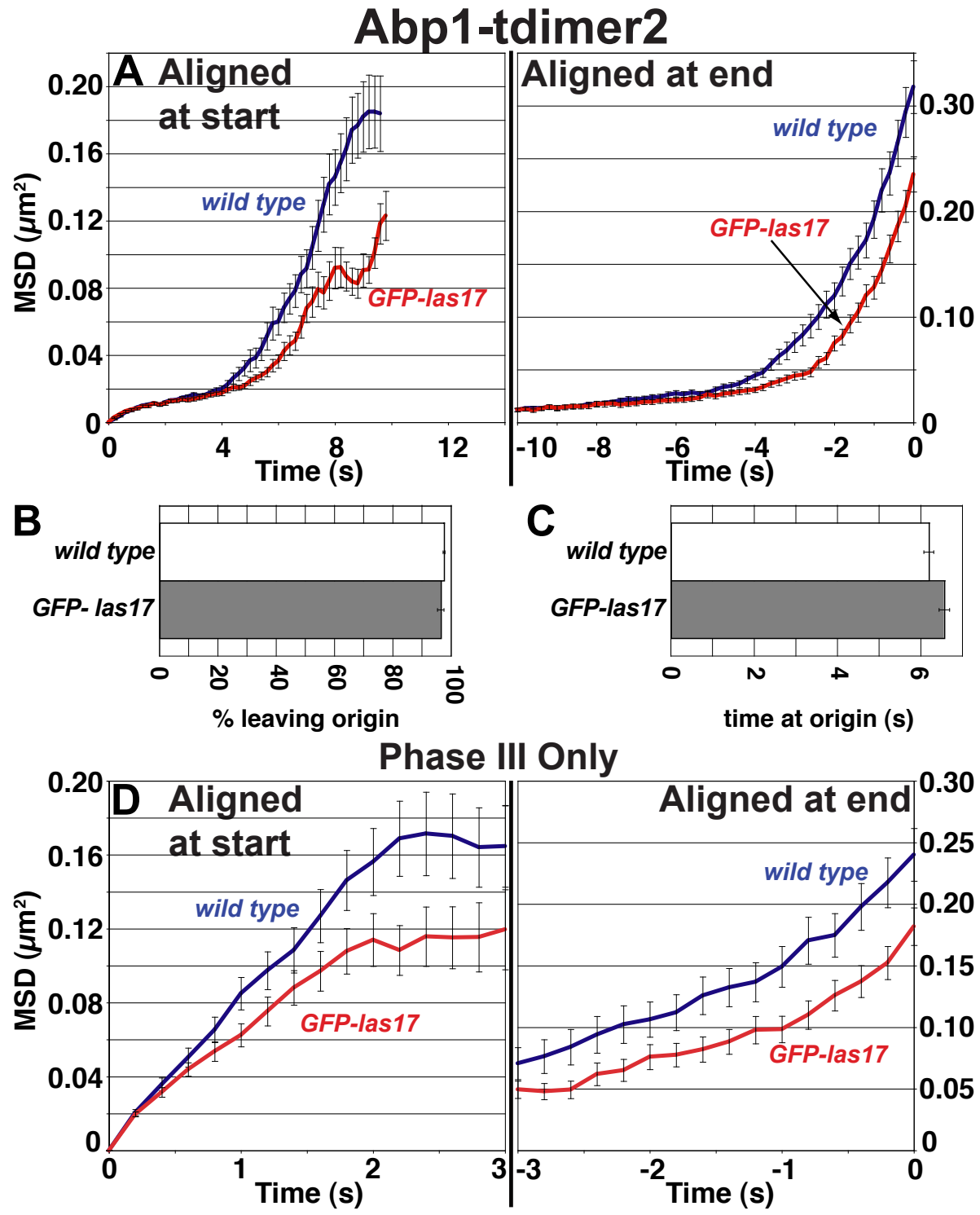
Abp1-tdimer2
A Aligned at start
Aligned at end
wild type
GFP-las17
MSD (μm²)
Time (s)
B
C
wild type
GFP- las17
GFP-las17
% leaving origin
time at origin (s)
Phase III Only
D Aligned at start
Aligned at end
