# Supplementary material for: Distinct Roles for Arp2/3 Regulators in Actin Assembly and Endocytosis
Source: PLoS Biol. 2008 Jan 3;6(1):e1. doi: 10.1371/journal.pbio.0060001 (PMC2156081; doi:10.1371/journal.pbio.0060001)
Supplement: Supplementary file 4 [file pbio.0060001.sg004.pdf]

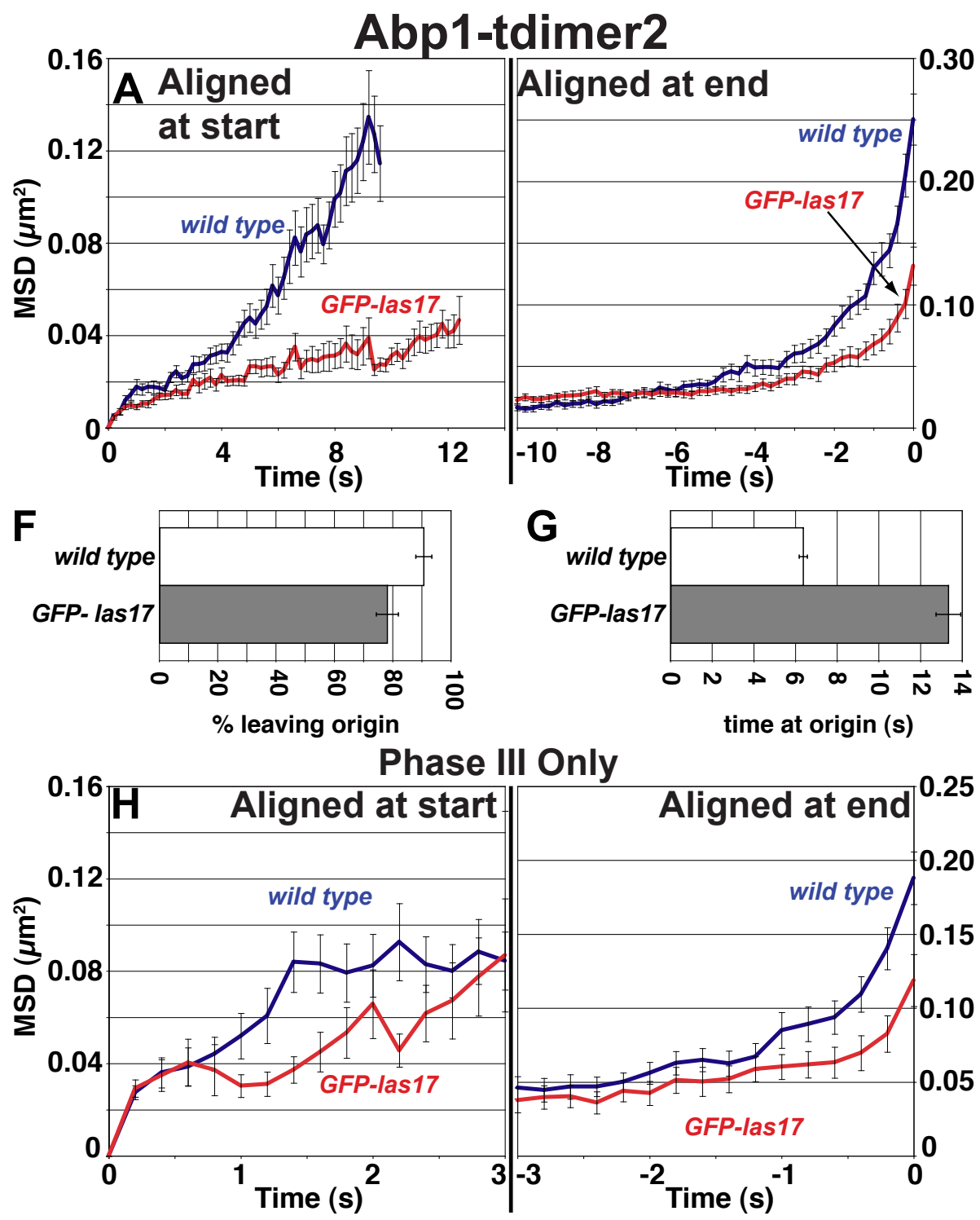

Abp1-tdimer2
A
Aligned at start
Aligned at end
MSD (µm²)
wild type
GFP-las17
Time (s)
F
wild type
GFP- las17
% leaving origin
G
wild type
GFP-las17
time at origin (s)
Phase III Only
H
Aligned at start
Aligned at end
MSD (µm²)
wild type
GFP-las17
Time (s)
