# Supplementary material for: Distinct Roles for Arp2/3 Regulators in Actin Assembly and Endocytosis
Source: PLoS Biol. 2008 Jan 3;6(1):e1. doi: 10.1371/journal.pbio.0060001 (PMC2156081; doi:10.1371/journal.pbio.0060001)
Supplement: Supplementary file 5 [file pbio.0060001.sd001.doc]

**Protocol S1**

**Strain construction**

*abp1∆, crn1∆, myo3∆* and *myo5∆* in a *MAT****a*** background and *aip1∆* and *crn1∆* in a *MATα* background were from the Yeast Gene Deletion Collection (Invitrogen, Carlsbad, CA, record numbers 7199, 6032, 4979, 6549, 16227 and 16032 respectively). *las17-GFP-HIS3* was from the Yeast GFP Collection (Invitrogen, Carlsbad, CA) YJC5695 was generated by crossing BY4741 and BY4742. YJC4338 was generated by crossing YJC2719 with BY4742. *las17∆acidic, myo3∆acidic, myo5∆acidic* and *PAN1-kanMX6* were generated by PCR mediated gene truncation in YJC4338 using pFA6a-GFP(S65T)-kanMX6 as a template [1]. F3 primers were utilized to exclude GFP and to only introduce a stop codon. YJC4616 was generated by introducing a stop codon and *kanMX6* after the codon encoding amino acid 608 of *las17*. YJC4617 was generated by introducing a stop codon and *kanMX6* after the codon for amino acid 1239 of *myo3*. YJC4620 was generated by introducing a stop codon and *kanMX6* after the codon for amino acid 1188 of *myo5*. YJC5697 was generated by introducing a new stop codon and *kanMX6* on top of the existing stop codon of *PAN1*, generating a wild-type *PAN1* marked with kanMX6. YJC4913 was generated by replacement of the *kanMX6* in YJC4616 with *CaUra3MX4* PCR amplified from pAG60 [2]. YJC4806 and YJC4681 were generated by replacement of the *kanMX6* in YJC4617 and *MAT****a*** *myo3∆* respectively with *hphMX4* with a PCR fragment amplified from pAG32 [3]. YJC4810 was generated by replacing *MYO5* in YJC4338 with *kanMX6* amplified by PCR from pFA6a-kanMX6 [1]. YJC5698 was generated using a mutagenic primer to remove the codons for amino acids 1259-1282 of *pan1* in YJC5695 by the method of Toulmay and Schneiter [4]. YJC5736 and YJC5737 were generated by transforming YJC5695 with a PCR product from pFA6a-HIS3MX6-PGAL1-GFP [1] to fuse GFP to the N-terminus of Las17 and place it under the control of the GAL1 promoter.

YJC4404 was generated from the tetrad dissection of a diploid generated by crossing YJC4336 and MATα myo5∆. YJC4915 was generated by tetrad dissection of YJC4913. YJC5204 was generated by crossing YJC4846 and YJC4915. YJC4808 was generated by crossing YJC4798 and YJC4796. YJC4837 – YJC4850 were generated by tetrad dissection of YJC4808. YJC4922 was generated by tetrad dissection of YJC4913. YJC5229 was generated from the cross of YJC3398 and YJC4922. YJC5231 – YJC5236 were generated by tetrad dissection of YJC5229. YJC5208 – 5210 and YJC5214 - 5216 were generated by tetrad dissection of YJC5204. YJC4846 was generated from the tetrad dissection of YJC4808. YJC4808 was generated from the cross of YJC4796 and YJC4798. YJC4796 was generated from the tetrad dissection of YJC4806. YJC4798 was generated from the tetrad dissection of 4620. YJC4784 – YJC4789 were generated by tetrad dissection of YJC4759. YJC4759 was generated by crossing YJC3398 with *MATα myo5∆*. YJC4813-YJC4817 were generated by tetrad dissection of YJC4810. YJC4409 – 4411 were generated from the tetrad dissection of a diploid generated by crossing YJC4336 and *MAT****a*** *myo3∆*. YJC4336 was generated from the tetrad dissection of a diploid generated by crossing YJC2719 and BY4742. YJC4770 – 4722 were generated from the tetrad dissection of YJC4761. YJC4761 was generated from the cross of YJC4681 x YJC4404. YJC5545 -YJC5568 were generated by tetrad dissection of YJC5543. YJC5543 was generated from the cross of YJC4846 and YJC5232. YJC4837-YJC4845, YJC4848 and YJC4849 were generated by tetrad dissection of YJC4808. YJC5205 – YJC5207, YJC5214 – YJC526, and YJC5438 – YJC5443 were generated by tetrad dissection of YJC5204. YJC4451 was generated by tetrad dissection of a diploid from the cross of YJC3398 and BY4742. YJC5452 was generated by crossing *MAT****a*** *abp1∆* and YJC4451. YJC5454-YJC5460 were generated by tetrad dissection of YJC5452. YJC5679 was generated from the cross of YJC2718 and YJC5460. YJC5681 – YJC5686 were generated by tetrad dissection of YJC5679.YJC5444 was generated by crossing *MAT****a*** *crn1∆* and YJC4451. YJC5446 – YJC5451 were generated by tetrad dissection of YJC5444. YJC4297-YJC4299 were generated by tetrad dissection of a diploid from the cross of YJC2719 and MATα aip1∆. YJC4235, YJC4236 and YJC4239 were generated by tetrad dissection of a cross between YJC2719 and *MATα crn1∆*. YJC5701 was generated by tetrad dissection of YJC5698. YJC5703 was generated by crossing YJC5701 and YJC4915. YJC5705 was generated by crossing YJC5701 and YJC5232. YJC5707 – YJC5718 were generated by tetrad dissection of YJC5705. YJC5719 – YJC5733 were generated by tetrad dissection of YJC5703.

YJC5756 was generated by transforming YJC5444 with PCR product to add tdimer2 to the c-terminus of Abp1 [5]. YJC5763 was derived from tetrad dissection of YJC5756. YJC6020 was generated by transforming YJC5792 was constructed by placing a HIS3 marker downstream of the coding sequence of Las17 in YJC5695 [1]. YJC5832 was constructed by PCR mediated replacement of 512 base pairs upstream of the ATG of *LAS17* with *URA3* in YJC5792. YJC5832 was transformed with a PCR product consisting of 554 base pairs upstream of the ATG of *las17* fused to GFP, which was then fused in frame to the coding sequence of *las17* and selecting for the loss of the *URA3* marker. This resulted in a N-terminally GFP tagged Las17 at the *las17* locus. DNA sequencing of this strain revealed a single nucleotide change of G to A, 239 base pairs upstream of the start of the translation start of the *gfp-las17* fusion. YJC6187 was generated by tetrad dissection of YJC6020. YJC6178 and YJC6179 were generated by crossing YJC5763 and YJC6187. YJC6180 – YJC6185 were generated by tetrad dissection of YJC6178.

YJC6039 was generated by crossing YJC5551 and YJC5763. YJC6041, YJC6043, YJC6044 and YJC6046 were generated by tetrad dissection of YJC6039. YJC5863 was generated from the cross of YJC5763 and BY4742. YJC5865 was generated from the tetrad dissection of YJC5863. YJC5950 – YJC5955 were generated by tetrad dissection of YJC5872. YJC5872 was generated by crossing YJC5865 with *LAS17-GFP-HIS3*. YJC5866 was generated by tetrad dissection of YJC5736. YJC5868 was generated YJC5868 was generated by crossing YJC5865 and YJC5866. YJC6021 – YJC6025 were generated by tetrad dissection of YJC5868.

1.	Longtine MS, McKenzie A, 3rd, Demarini DJ, Shah NG, Wach A, et al. (1998) Additional modules for versatile and economical PCR-based gene deletion and modification in Saccharomyces cerevisiae. Yeast 14: 953-961.

2.	Goldstein AL, Pan X, McCusker JH (1999) Heterologous URA3MX cassettes for gene replacement in Saccharomyces cerevisiae. Yeast 15: 507-511.

3.	Goldstein AL, McCusker JH (1999) Three new dominant drug resistance cassettes for gene disruption in Saccharomyces cerevisiae. Yeast 15: 1541-1553.

4.	Toulmay A, Schneiter R (2006) A two-step method for the introduction of single or multiple defined point mutations into the genome of Saccharomyces cerevisiae. Yeast 23: 825-831.

5.	Sheff MA, Thorn KS (2004) Optimized cassettes for fluorescent protein tagging in Saccharomyces cerevisiae. Yeast 21: 661-670.
