# Supplementary material for: Distinct Roles for Arp2/3 Regulators in Actin Assembly and Endocytosis
Source: PLoS Biol. 2008 Jan 3;6(1):e1. doi: 10.1371/journal.pbio.0060001 (PMC2156081; doi:10.1371/journal.pbio.0060001)
Supplement: Supplementary file 6 [file pbio.0060001.st001.doc]

| **Strain** | **Genotype** | **Source** |
| --- | --- | --- |
| BY4741 | *MAT****a*** *his3∆1 leu2∆0 met15∆0 ura3∆0* | [1] |
| BY4742 | *MATα his3∆1 leu2∆0 lys2∆0 ura3∆0* | [1] |
| YJC2718 | *MAT****a*** *CAP1-GFP-HIS3 his3∆1 leu2∆0 met15∆0 ura3∆0* | [2] |
| YJC2719 | *MAT****a*** *ABP1-GFP-HIS3 his3∆1 leu2∆0 met15∆0 ura3∆0* | [3] |
| YJC3398 | *MAT****a*** *SLA2-GFP-HIS3 his3∆1 leu2∆0 met15∆0 ura3∆0* | [3] |
| YJC4235 | *MAT****a*** *crn1∆::kanMX4 ABP1-GFP-HIS3 his3∆1 leu2∆0 met15∆0 ura3∆0* | This study |
| YJC4236 | *MATα crn1∆::kanMX4 ABP1-GFP-HIS3 his3∆1 leu2∆0 lys2∆0 met15∆0 ura3∆0* | This study |
| YJC4239 | *MAT****a*** *crn1∆::kanMX4 ABP1-GFP-HIS3 his3∆1 leu2∆0 lys2∆0 ura3∆0* | This study |
| YJC4297 | *MATα ABP1-GFP-HIS3 his3∆1 leu2∆0 ura3∆0* | This study |
| YJC4298 | *MAT****a*** *ABP1-GFP-HIS3 his3∆1 leu2∆0 ura3∆0* | This study |
| YJC4299 | *MAT****a*** *ABP1-GFP-HIS3 his3∆1 leu2∆0 met15∆0 ura3∆0* | This study |
| YJC4336 | *MAT****a*** *ABP1-GFP-HIS3 his3∆1 leu2∆0 met15∆0 ura3∆0* | This study |
| YJC4338 | *MATα/MAT****a*** *ABP1-GFP-HIS3/+ his3∆1/his3∆1 leu2∆0/leu2∆0 lys2∆0/LYS2 met15∆0/MET15 ura3∆0/ura3∆0* | This study |
| YJC4404 | *MAT****a*** *myo5∆::kanMX4 ABP1-GFP-HIS3 his3∆1 leu2∆0 met15∆0 ura3∆0* | This study |
| YJC4409 | *MATα ABP1-GFP-HIS3 myo3∆::kanMX4 his3∆1 leu2∆0 met15∆0 ura3∆0* | This study |
| YJC4410 | *MAT****a*** *ABP1-GFP-HIS3 myo3∆::kanMX4 his3∆1 leu2∆0 met15∆0 ura3∆0* | This study |
| YJC4411 | *MAT****a*** *ABP1-GFP-HIS3 myo3∆::kanMX4 his3∆1 leu2∆0 met15∆0 ura3∆0* | This study |
| YJC4451 | *MATα SLA2-GFP-HIS3 his3∆1 leu2∆0 lys2∆0 ura3∆0* | This study |
| YJC4616 | *MATα/MAT****a*** *las17∆acidic-kanMX6/LAS17 ABP1-GFP-HIS3/ABP1 his3∆1/his3∆1 leu2∆0/leu2∆0 lys2∆0/LYS2 met15∆0/MET15 ura3∆0/ura3∆0* | This study |
| YJC4617 | *MATα/MAT****a*** *myo3∆acidic-kanMX6/MYO3 ABP1-GFP-HIS3/ABP1 his3∆1/his3∆1 leu2∆0/leu2∆0 lys2∆0/LYS2 met15∆0/MET15 ura3∆0/ura3∆0* | This study |
| YJC4620 | *MATα/MAT****a*** *myo5∆acidic-kanMX6/MYO5 ABP1-GFP-HIS3/ABP1 his3∆1/his3∆1 leu2∆0/leu2∆0 lys2∆0/LYS2 met15∆0/MET15 ura3∆0/ura3∆0* | This study |
| YJC4681 | *MATα myo3∆::hphMX4 his3∆1 leu2∆0 lys2∆0 ura3∆0* | This study |
| YJC4759 | *MATα/MAT****a*** *myo5∆::kanMX4/MYO5 SLA2-GFP-HIS3/SLA2 his3∆1/his3∆1 leu2∆0/leu2∆0 lys2∆0/LYS2 met15∆0/MET15 ura3∆0/ura3∆0* | This study |
| YJC4761 | *MATα/MAT****a*** *myo3∆::hphMX4/MYO3 myo5∆::kanMX4/MYO5 ABP1-GFP-HIS3/ABP1 his3∆1/his3∆1 leu2∆0/leu2∆0 lys2∆0/LYS2 met15∆0/MET15 ura3∆0/ura3∆0* | This study |
| YJC4770 | *MAT****a*** *myo3∆::hphMX4 myo5∆::kanMX4 ABP1-GFP-HIS3 his3∆1 leu2∆0 lys2∆0 ura3∆0* | This study |
| YJC4771 | *MATα myo3∆::hphMX4 myo5∆::kanMX4 ABP1-GFP-HIS3 his3∆1 leu2∆0 met15∆0 ura3∆0* | This study |
| YJC4772 | *MAT****a*** *myo3∆::hphMX4 myo5∆::kanMX4 ABP1-GFP-HIS3 his3∆1 leu2∆0 met15∆0 ura3∆0* | This study |
| YJC4784 | *MATα myo5∆::kanMX4 SLA2-GFP-HIS3 his3∆1 leu2∆0 lys2∆0 ura3∆0* | This study |
| YJC4785 | *MAT****a*** *myo5∆::kanMX4 SLA2-GFP-HIS3 his3∆1 leu2∆0 lys2∆0 ura3∆0* | This study |
| YJC4786 | *MAT****a*** *myo5∆::kanMX4 SLA2-GFP-HIS3 his3∆1 leu2∆0 lys2∆0 met15∆0 ura3∆0* | This study |
| YJC4787 | *MAT****a*** *SLA2-GFP-HIS3 his3∆1 leu2∆0 ura3∆0* | This study |
| YJC4788 | *MATα SLA2-GFP-HIS3 his3∆1 leu2∆0 lys2∆0 ura3∆0* | This study |
| YJC4789 | *MAT****a*** *SLA2-GFP-HIS3 his3∆1 leu2∆0 lys2∆0 ura3∆0* | This study |
| YJC4796 | *MAT****a*** *myo3∆acidic-hphMX4 his3∆1 leu2∆0 met15∆0 ura3∆0* | This study |
| YJC4798 | *MATα myo5∆acidic-kanMX6 ABP1-GFP-HIS3 his3∆1 leu2∆0 lys2∆0 ura3∆0* | This study |
| YJC4806 | *MATα/MAT****a*** *myo3∆acidic-hphMX4/MYO3 ABP1-GFP-HIS3/ABP1 his3∆1/his3∆1 leu2∆0/leu2∆0 lys2∆0/LYS2 met15∆0/MET15 ura3∆0/ura3∆0* | This study |
| YJC4808 | *MATα/MAT****a*** *myo3∆acidic-hphMX4/MYO3 myo5∆acidic-kanMX6/MYO5 ABP1-GFP-HIS3/ABP1 his3∆1/his3∆1 leu2∆0/leu2∆0 lys2∆0/LYS2 met15∆0/MET15 ura3∆0/ura3∆0* | This study |
| YJC4810 | *MATα/MAT****a*** *myo5∆::kanMX6/MYO5 ABP1-GFP-HIS3/ABP1 his3∆1/his3∆1 leu2∆0/leu2∆0 lys2∆0/LYS2 met15∆0/MET15 ura3∆0/ura3∆0* | This study |
| YJC4813 | *MAT****a*** *myo5∆::kanMX6 ABP1-GFP-HIS3 his3∆1 leu2∆0 ura3∆0* | This study |
| YJC4814 | *MAT****a*** *myo5∆::kanMX6 ABP1-GFP-HIS3 his3∆1 leu2∆0 ura3∆0* | This study |
| YJC4815 | *MAT****a*** *ABP1-GFP-HIS3 his3∆1 leu2∆0 met15∆0 ura3∆0* | This study |
| YJC4816 | *MATα ABP1-GFP-HIS3 his3∆1 leu2∆0 lys2∆0 ura3∆0* | This study |
| YJC4817 | *MATα ABP1-GFP-HIS3 his3∆1 leu2∆0 lys2∆0 ura3∆0* | This study |
| YJC4837 | *MAT****a*** *myo3∆acidic-hphMX4 myo5∆acidic-kanMX6 ABP1-GFP-HIS3 his3∆1 leu2∆0 met15∆0 ura3∆0* | This study |
| YJC4837 | *MAT****a*** *myo3∆acidic-hphMX4 myo5∆acidic-kanMX6 ABP1-GFP-HIS3 his3∆1 leu2∆0 met15∆0 ura3∆0* | This study |
| YJC4838 | *MAT****a*** *myo3∆acidic-hphMX4 ABP1-GFP-HIS3 myo5∆acidic-kanMX6 his3∆1 leu2∆0 lys2∆0 met15∆0 ura3∆0* | This study |
| YJC4838 | *MAT****a*** *myo3∆acidic-hphMX4 myo5∆acidic-kanMX6 ABP1-GFP-HIS3 his3∆1 leu2∆0 lys2∆0 met15∆0 ura3∆0* | This study |
| YJC4839 | *MATα myo3∆acidic-hphMX4 ABP1-GFP-HIS3 myo5∆acidic-kanMX6 his3∆1 leu2∆0 lys2∆0 ura3∆0* | This study |
| YJC4839 | *MATα myo3∆acidic-hphMX4 myo5∆acidic-kanMX6 ABP1-GFP-HIS3 his3∆1 leu2∆0 lys2∆0 ura3∆0* | This study |
| YJC4840 | *MAT****a*** *myo5∆acidic-kanMX6 ABP1-GFP-HIS3 his3∆1 leu2∆0 lys2∆0 met15∆0 ura3∆0* | This study |
| YJC4840 | *MAT****a*** *myo5∆acidic-kanMX6 ABP1-GFP-HIS3 his3∆1 leu2∆0 lys2∆0 met15∆0 ura3∆0* | This study |
| YJC4841 | *MATα myo5∆acidic-kanMX6 ABP1-GFP-HIS3 his3∆1 leu2∆0 met15∆0 ura3∆0* | This study |
| YJC4841 | *MATα myo5∆acidic-kanMX6 ABP1-GFP-HIS3 his3∆1 leu2∆0 met15∆0 ura3∆0* | This study |
| YJC4842 | *MATα myo5∆acidic-kanMX6 ABP1-GFP-HIS3 his3∆1 leu2∆0 lys2∆0 ura3∆0* | This study |
| YJC4842 | *MATα myo5∆acidic-kanMX6 ABP1-GFP-HIS3 his3∆1 leu2∆0 lys2∆0 ura3∆0* | This study |
| YJC4843 | *MATα myo3∆acidic-hphMX4 ABP1-GFP-HIS3 his3∆1 leu2∆0 lys2∆0 met15∆0 ura3∆0* | This study |
| YJC4843 | *MATα myo3∆acidic-hphMX4 ABP1-GFP-HIS3 his3∆1 leu2∆0 lys2∆0 met15∆0 ura3∆0* | This study |
| YJC4844 | *MAT****a*** *myo3∆acidic-hphMX4 ABP1-GFP-HIS3 his3∆1 leu2∆0 met15∆0 ura3∆0* | This study |
| YJC4844 | *MAT****a*** *myo3∆acidic-hphMX4 ABP1-GFP-HIS3 his3∆1 leu2∆0 met15∆0 ura3∆0* | This study |
| YJC4845 | *MATα myo3∆acidic-hphMX4 ABP1-GFP-HIS3 his3∆1 leu2∆0 met15∆0 ura3∆0* | This study |
| YJC4845 | *MATα myo3∆acidic-hphMX4 ABP1-GFP-HIS3 his3∆1 leu2∆0 met15∆0 ura3∆0* | This study |
| YJC4846 | *MAT****a*** *myo3∆acidic/DDW-hphMX4 myo5∆acidic-kanMX6 his3∆1 leu2∆0 met15∆0 ura3∆0* | This study |
| YJC4848 | *MATα ABP1-GFP-HIS3 his3∆1 leu2∆0 lys2∆0 ura3∆0* | This study |
| YJC4848 | *MATα ABP1-GFP-HIS3 his3∆1 leu2∆0 lys2∆0 ura3∆0* | This study |
| YJC4849 | *MATα ABP1-GFP-HIS3 his3∆1 leu2∆0 lys2∆0 met15∆0 ura3∆0* | This study |
| YJC4850 | *MAT****a*** *ABP1-GFP-HIS3 his3∆1 leu2∆0met15∆0 ura3∆0* | This study |
| YJC4913 | *MATα/MAT****a*** *las17∆acidic-CaURA3MX4/LAS17 ABP1-GFP-HIS3/ABP1 his3∆1/his3∆1 leu2∆0/leu2∆0 lys2∆0/LYS2 met15∆0/MET15 ura3∆0/ura3∆0* | This study |
| YJC4915 | *MATα las17∆acidic-CaURA3MX4 ABP1-GFP-HIS3 his3∆1 lys2∆0 leu2∆0 ura3∆0* | This study |
| YJC4922 | *MATα las17∆acidic-CaURA3MX4 his3∆1 leu2∆0 lys2∆0 ura3∆0* | This study |
| YJC5204 | *MATα/MAT****a*** *ABP1-GFP-HIS3/ABP1 las17∆acidic-CaURA3MX4/LAS17 myo3∆acidic-hphMX4/MYO3 myo5∆acidic-kanMX6/MYO5 his3∆1/his3∆1 leu2∆0/leu2∆0 lys2∆0/LYS2 met15∆0/MET15 ura3∆0/ura3∆0* | This study |
| YJC5205 | *MATα las17∆acidic-CaURA3MX4 myo3∆acidic-hphMX4 myo5∆acidic-kanMX6 ABP1-GFP-HIS3 his3∆1 leu2∆0 met15∆0 ura3∆0* | This study |
| YJC5206 | *MAT****a*** *las17∆acidic-CaURA3MX4 myo3∆acidic-hphMX4 myo5∆acidic-kanMX6 ABP1-GFP-HIS3 his3∆1 leu2∆0 met15∆0 ura3∆0* | This study |
| YJC5208 | *MATα las17∆acidic-CaURA3MX4 ABP1-GFP-HIS3 his3∆1 leu2∆0 ura3∆0* | This study |
| YJC5209 | *MAT****a*** *las17∆acidic-CaURA3MX4 ABP1-GFP-HIS3 his3∆1 leu2∆0 ura3∆0* | This study |
| YJC5210 | *MATα las17∆acidic-CaURA3MX4 ABP1-GFP-HIS3 his3∆1 leu2∆0 lys2∆0 ura3∆0* | This study |
| YJC5214 | *MATα ABP1-GFP-HIS3 his3∆1 leu2∆0 lys2∆0 met15∆0 ura3∆0* | This study |
| YJC5214 | *MATα ABP1-GFP-HIS3 his3∆1 leu2∆0 lys2∆0 met15∆0 ura3∆0* | This study |
| YJC5215 | *MATα ABP1-GFP-HIS3 his3∆1 leu2∆0 lys2∆0 met15∆0 ura3∆0* | This study |
| YJC5215 | *MATα ABP1-GFP-HIS3 his3∆1 leu2∆0 lys2∆0 met15∆0 ura3∆0* | This study |
| YJC5216 | *MAT****a*** *ABP1-GFP-HIS3 his3∆1 leu2∆0 lys2∆0 met15∆0 ura3∆0* | This study |
| YJC5216 | *MAT****a*** *ABP1-GFP-HIS3 his3∆1 leu2∆0 lys2∆0 met15∆0 ura3∆0* | This study |
| YJC5229 | *MATα/MATa las17∆acidic-CaURA3MX4/LAS17 SLA2-GFP-HIS3/SLA2 his3∆1/his3∆1 leu2∆0/leu2∆0 lys2∆0/LYS2 met15∆0/MET15 ura3∆0/ura3∆0* | This study |
| YJC5231 | *MAT****a*** *las17∆acidic-CaURA3MX4 SLA2-GFP-HIS3 his3∆1 leu2∆0 met15∆0 ura3∆0* | This study |
| YJC5232 | *MATα las17∆acidic-CaURA3MX4 SLA2-GFP-HIS3 his3∆1 leu2∆0 lys2∆0 ura3∆0* | This study |
| YJC5233 | *MAT****a*** *las17∆acidic-CaURA3MX4 SLA2-GFP-HIS3 his3∆1 leu2∆0 met15∆0 ura3∆0* | This study |
| YJC5234 | *MAT****a*** *SLA2-GFP-HIS3 his3∆1 leu2∆0 lys2∆0 ura3∆0* | This study |
| YJC5235 | *MAT****a*** *SLA2-GFP-HIS3 his3∆1 leu2∆0 ura3∆0* | This study |
| YJC5236 | *MATα SLA2-GFP-HIS3 his3∆1 leu2∆0 lys2∆0 ura3∆0* | This study |
| YJC5438 | *MAT****a*** *las17∆acidic-URAMX4 myo3∆acidic-hphMX ABP1-GFP-HIS3 his3∆1 leu2∆0 lys2∆0 met15∆0 ura3∆0* | This study |
| YJC5439 | *MATα las17∆acidic-URAMX4 myo3∆acidic-hphMX ABP1-GFP-HIS3 his3∆1 leu2∆0 lys2∆0 ura3∆0* | This study |
| YJC5440 | *MATα* *las17∆acidic-URAMX4 myo3∆acidic-hphMX ABP1-GFP-HIS3 his3∆1 leu2∆0 met15∆ ura3∆0* | This study |
| YJC5441 | *MAT****a*** *las17∆acidic-URAMX4 myo5∆acidic-kanMX ABP1-GFP-HIS3 his3∆1 leu2∆0 ura3∆0* | This study |
| YJC5442 | *MATα las17∆acidic-URAMX4 myo5∆acidic-kanMX ABP1-GFP-HIS3 his3∆1 leu2∆0 lys2∆0 met15∆ ura3∆0* | This study |
| YJC5443 | *MAT****a*** *las17∆acidic-URAMX4 myo5∆acidic-kanMX ABP1-GFP-HIS3 his3∆1 leu2∆0 lys2∆0 ura3∆0* | This study |
| YJC5444 | *MATα/MAT****a*** *crn1∆::KanMX4/CRN1 SLA2-GFP-HIS/SLA2 his3∆1/his3∆1 leu2∆0/leu2∆0 lys2∆0/LYS2 met15∆0/MET15 ura3∆0/ura3∆0* | This study |
| YJC5446 | *MATα crn1∆::kanMX4 SLA2-GFP-HIS3 his3∆1 leu2∆0 lys2∆0 ura3∆0* | This study |
| YJC5447 | *MAT****a*** *crn1∆::kanMX4 SLA2-GFP-HIS3 his3∆1 leu2∆0 ura3∆0* | This study |
| YJC5448 | *MATα crn1∆::kanMX4 SLA2-GFP-HIS3 his3∆1 leu2∆0 lys2∆0 ura3∆0* | This study |
| YJC5449 | *MAT****a*** *SLA2-GFP-HIS3 his3∆1 leu2∆0 lys2∆0 met15∆0 ura3∆0* | This study |
| YJC5450 | *MAT****a*** *SLA2-GFP-HIS3 his3∆1 leu2∆0 met15∆0 ura3∆0* | This study |
| YJC5451 | *MATα SLA2-GFP-HIS3 his3∆1 leu2∆0 met15∆0 ura3∆0* | This study |
| YJC5452 | *MATα/MAT****a*** *abp1∆::kanMX4/ABP1 SLA2-GFP-HIS3/SLA2 his3∆1/his3∆1 leu2∆0/leu2∆0 lys2∆0/LYS2 met15∆0/MET15 ura3∆0/ura3∆0* | This study |
| YJC5454 | *MATα abp1∆::kanMX4 SLA2-GFP-HIS3 his3∆1 leu2∆0 lys2∆0 ura3∆0* | This study |
| YJC5455 | *MAT****a*** *abp1∆::kanMX4 SLA2-GFP-HIS3 his3∆1 leu2∆0 met15∆0 ura3∆0* | This study |
| YJC5456 | *MAT****a*** *abp1∆::kanMX4 SLA2-GFP-HIS3 his3∆1 leu2∆0 lys2∆0 ura3∆0* | This study |
| YJC5457 | *MAT****a*** *SLA2-GFP-HIS3 his3∆1 leu2∆0 met15∆0 ura3∆0* | This study |
| YJC5458 | *MATα SLA2-GFP-HIS3 his3∆1 leu2∆0 met15∆0 lys2∆0 ura3∆0* | This study |
| YJC5459 | *MATα SLA2-GFP-HIS3 his3∆1 leu2∆0 lys2∆0 ura3∆0* | This study |
| YJC5460 | *MATα abp1∆::kanMX4 his3∆1 leu2∆0 lys2∆0 ura3∆0* | This study |
| YJC5543 | *MATα/MAT****a*** *las17∆acidic-uraMX4/LAS17 myo3∆acidic-hphMX4/MYO3 myo5∆acidic-kanMX6/MYO5 SLA2-GFP-HIS3/SLA2 his3∆1/his3∆1 leu2∆0/leu2∆0 met15∆0/MET15 lys2∆0/LYS2 ura3∆0/ura3∆0* | This study |
| YJC5545 | *MATα las17∆acidic-uraMX4 myo3∆acidic-hphMX4 myo5∆acidic-KanMX6 SLA2-GFP-HIS3 his3∆1 leu2∆0 lys2∆0 ura3∆0* | This study |
| YJC5546 | *MAT****a*** *las17∆acidic-uraMX4 myo3∆acidic-hphMX4 myo5∆acidic-kanMX6 SLA2-GFP-HIS3 his3∆1 leu2∆0 lys2∆0 met15∆0 ura3∆0* | This study |
| YJC5547 | *MAT****a*** *las17∆acidic-uraMX4 myo3∆acidic-hphMX4 myo5∆acidic-kanMX6 SLA2-GFP-HIS3 his3∆1 leu2∆0 ura3∆0* | This study |
| YJC5548 | *MAT****a*** *las17∆acidic-uraMX4 myo5∆acidic-kanMX6 SLA2-GFP-HIS3 his3∆1 leu2∆0 ura3∆0* | This study |
| YJC5549 | *MATα las17∆acidic-uraMX4 myo5∆acidic-kanMX6 SLA2-GFP-HIS3 his3∆1 leu2∆0 ura3∆0* | This study |
| YJC5550 | *MAT****a*** *las17∆acidic-uraMX4 myo5∆acidic-KanMX6 SLA2-GFP-HIS3 his3∆1 leu2∆0 met15∆0 lys2∆0 ura3∆0* | This study |
| YJC5551 | *MATα las17∆acidic-uraMX4 SLA2-GFP-HIS3 his3∆1 leu2∆0 lys2∆0 ura3∆0* | This study |
| YJC5552 | *MAT****a*** *las17∆acidic-uraMX4 SLA2-GFP-HIS3 his3∆1 leu2∆0 met15∆0 lys2∆0 ura3∆0* | This study |
| YJC5553 | *MATα las17∆acidic-uraMX4 SLA2-GFP-HIS3 his3∆1 leu2∆0 lys2∆0 ura3∆0* | This study |
| YJC5554 | *MATα myo5∆acidic-kanMX6 SLA2-GFP-HIS3 his3∆1 leu2∆0 met15∆0 ura3∆0* | This study |
| YJC5555 | *MAT****a*** *myo5∆acidic-kanMX6 SLA2-GFP-HIS3 his3∆1 leu2∆0 ura3∆0* | This study |
| YJC5556 | *MATα myo5∆acidic-kanMX6 SLA2-GFP-HIS3 his3∆1 leu2∆0 lys2∆0 met15∆0 ura3∆0* | This study |
| YJC5557 | *MATα myo3∆acidic-hphMX4 myo5∆acidic-kanMX6 SLA2-GFP-HIS3 his3∆1 leu2∆0 lys2∆0 met15∆0 ura3∆0* | This study |
| YJC5558 | *MATα myo3∆acidic-hphMX4 myo5∆acidic-kanMX6 SLA2-GFP-HIS3 his3∆1 leu2∆0 lys2∆0 met15∆0 ura3∆0* | This study |
| YJC5559 | *MATα* *myo3∆acidic-hphMX4 myo5∆acidic-kanMX6 SLA2-GFP-HIS3 his3∆1 leu2∆0 ura3∆0* | This study |
| YJC5560 | *MAT****a*** *las17∆acidic-uraMX4 myo3∆acidic-hphMX4 SLA2-GFP-HIS3 his3∆1 leu2∆0 ura3∆0* | This study |
| YJC5561 | *MAT****a*** *las17∆acidic-uraMX4 myo3∆acidic-hphMX4 SLA2-GFP-HIS3 his3∆1 leu2∆0 met15∆0 ura3∆0* | This study |
| YJC5562 | *MATα* *las17∆acidic-uraMX4 myo3∆acidic-hphMX4 SLA2-GFP-HIS3 his3∆1 leu2∆0 lys2∆0 ura3∆0* | This study |
| YJC5563 | *MAT****a*** *myo3∆acidic-hphMX4 SLA2-GFP-HIS3 his3∆1 leu2∆0 lys2∆0 met15∆0 ura3∆0* | This study |
| YJC5564 | *MATα myo3∆acidic-hphMX4 SLA2-GFP-HIS3 his3∆1 leu2∆0 ura3∆0* | This study |
| YJC5565 | *MAT****a*** *myo3∆acidic-hphMX4 SLA2-GFP-HIS3 his3∆1 leu2∆0 met15∆0 ura3∆0* | This study |
| YJC5566 | *MATα SLA2-GFP-HIS3 his3∆1 leu2∆0 lys2∆0 ura3∆0* | This study |
| YJC5567 | *MATα SLA2-GFP-HIS3 his3∆1 leu2∆0 lys2∆0 met15∆0 ura3∆0* | This study |
| YJC5568 | *MATα SLA2-GFP-HIS3 his3∆1 leu2∆0 met15∆0 ura3∆0* | This study |
| YJC5679 | *MATα/MAT****a*** *abp1∆::kanMX4/ABP1 CAP1-GFP-HIS3/CAP1 his3∆1/his3∆1 leu2∆0/leu2∆0 lys2∆0 /LYS2 met15∆0/MET15 ura3∆0/ura3∆0* | This study |
| YJC5681 | *MATα CAP1-GFP-HIS3 his3∆1 leu2∆0 met15∆0 ura3∆0* | This study |
| YJC5682 | *MATα CAP1-GFP-HIS3 his3∆1 leu2∆0 ura3∆0* | This study |
| YJC5683 | *MAT****a*** *CAP1-GFP-HIS3 his3∆1 leu2∆0 lys2∆0 ura3∆0* | This study |
| YJC5684 | *MAT****a*** *abp1∆::kanMX4 CAP1-GFP-HIS3 his3∆1 leu2∆0 ura3∆0* | This study |
| YJC5685 | *MATα abp1∆::kanMX4 CAP1-GFP-HIS3 his3∆1 leu2∆0 lys2∆0 ura3∆0* | This study |
| YJC5686 | *MATα abp1∆::kanMX4 CAP1-GFP-HIS3 his3∆1 leu2∆0 met15∆0 ura3∆0* | This study |
| YJC5695 | *MATα/MAT****a*** *his3∆1/his3∆1 leu2∆0/leu2∆0 lys2∆0/LYS2 met15∆0/MET15 ura3∆0/ura3∆0* | This study |
| YJC5697 | *MATα/MAT****a*** *PAN1-kanMX6/PAN1 his3∆1/his3∆1 leu2∆0/leu2∆0 lys2∆0/LYS2 met15∆0/MET15 ura3∆0/ura3∆0* | This study |
| YJC5698 | *MATα/MAT****a*** *pan1∆acidic-kanMX6/PAN1 his3∆1/his3∆1 leu2∆0/leu2∆0 lys2∆0/LYS2 met15∆0/MET15 ura3∆0/ura3∆0* | This study |
| YJC5701 | *MAT****a*** *pan1∆acidic-kanMX6 his3∆1 leu2∆0 met15∆0 ura3∆0* | This study |
| YJC5703 | *MATα/MAT****a*** *las17∆acidic-CaURA3MX4/LAS17 pan1∆acidic-kanMX6/PAN1 ABP1-GFP-HIS3/ABP1 his3∆1/ his3∆1 leu2∆0/leu2∆0 lys2∆0/LYS2 met15∆0/MET15 ura3∆0/ura3∆0* | This study |
| YJC5705 | *MATα/MAT****a*** *las17∆acidic-CaURA3MX4/LAS17 pan1∆acidic-kanMX6/PAN1 SLA2-GFP-HIS3/SLA2 his3∆1/ his3∆1 leu2∆0/leu2∆0 lys2∆0/LYS2 met15∆0/MET15 ura3∆0/ura3∆0* | This study |
| YJC5707 | *MATα SLA2-GFP-HIS3 his3∆1 leu2∆0 met15∆0 ura3∆0* | This study |
| YJC5708 | *MAT****a*** *SLA2-GFP-HIS3 his3∆1 leu2∆0 met15∆0 ura3∆0* | This study |
| YJC5709 | *MATα SLA2-GFP-HIS3 his3∆1 leu2∆0 lys2∆0 ura3∆0* | This study |
| YJC5710 | *MATα pan1∆acidic-kanMX6 SLA2-GFP-HIS3 his3∆1 leu2∆0 lys2∆0 ura3∆0* | This study |
| YJC5711 | *MAT****a*** *pan1∆acidic-kanMX6 SLA2-GFP-HIS3 his3∆1 leu2∆0 met15∆0 ura3∆0* | This study |
| YJC5712 | *MATα pan1∆acidic-kanMX6 SLA2-GFP-HIS3 his3∆1 leu2∆0 lys2∆0 met15∆0 ura3∆0* | This study |
| YJC5713 | *MAT****a*** *las17∆acidic-CaURA3MX4 SLA2-GFP-HIS3 his3∆1 leu2∆0 met15∆0 ura3∆0* | This study |
| YJC5714 | *MATα las17∆acidic-CaURA3MX4 SLA2-GFP-HIS3 his3∆1 leu2∆0 lys∆0 ura3∆0* | This study |
| YJC5715 | *MATα las17∆acidic-CaURA3MX4 SLA2-GFP-HIS3 his3∆1 leu2∆0 lys∆0 met15∆0 ura3∆0* | This study |
| YJC5716 | *MAT****a*** *las17∆acidic-CaURA3MX4 pan1∆acidic-kanMX6 SLA2-GFP-HIS3 his3∆1 leu2∆0 met15∆0 ura3∆0* | This study |
| YJC5717 | *MATα las17∆acidic-CaURA3MX4 pan1∆acidic-kanMX6 SLA2-GFP-HIS3 his3∆1 leu2∆0 lys2∆0 ura3∆0* | This study |
| YJC5718 | *MAT****a*** *las17∆acidic-CaURA3MX4 pan1∆acidic-kanMX6 SLA2-GFP-HIS3 his3∆1 leu2∆0 lys2∆0 ura3∆0* | This study |
| YJC5719 | *MATα ABP1-GFP-HIS3 his3∆1 leu2∆0 lys2∆0 ura3∆0* | This study |
| YJC5720 | *MAT****a*** *ABP1-GFP-HIS3 his3∆1 leu2∆0 ura3∆0* | This study |
| YJC5721 | *MAT****a*** *ABP1-GFP-HIS3 his3∆1 leu2∆0 lys2∆0 ura3∆0* | This study |
| YJC5722 | *MATα ABP1-GFP-HIS3 his3∆1 leu2∆0 lys2∆0 met15∆0 ura3∆0* | This study |
| YJC5723 | *MAT****a*** *pan1∆acidic-kanMX6 ABP1-GFP-HIS3 his3∆1 leu2∆0 met15∆0 ura3∆0* | This study |
| YJC5724 | *MATα pan1∆acidic-kanMX6 ABP1-GFP-HIS3 his3∆1 leu2∆0 lys2∆0 ura3∆0* | This study |
| YJC5725 | *MATα pan1∆acidic-kanMX6 ABP1-GFP-HIS3 his3∆1 leu2∆0 lys2∆0 ura3∆0* | This study |
| YJC5726 | *MATα las17∆acidic-CaURA3MX4 ABP1-GFP-HIS3 his3∆1 leu2∆0 met15∆0 lys∆0 ura3∆0* | This study |
| YJC5727 | *MAT****a*** *las17∆acidic-CaURA3MX4 ABP1-GFP-HIS3 his3∆1 leu2∆0 ura3∆0* | This study |
| YJC5728 | *MAT****a*** *las17∆acidic-CaURA3MX4 ABP1-GFP-HIS3 his3∆1 leu2∆0 met15∆0 ura3∆0* | This study |
| YJC5729 | *MATα las17∆acidic-CaURA3MX4 ABP1-GFP-HIS3 his3∆1 leu2∆0 met15∆0 ura3∆0* | This study |
| YJC5730 | *MAT****a*** *las17∆acidic-CaURA3MX4 pan1∆acidic-kanMX6 ABP1-GFP-HIS3 his3∆1 leu2∆0 met15∆0 ura3∆0* | This study |
| YJC5731 | *MATα las17∆acidic-CaURA3MX4 pan1∆acidic-kanMX6 ABP1-GFP-HIS3 his3∆1 leu2∆0 lys2∆0 ura3∆0* | This study |
| YJC5732 | *MATα las17∆acidic-CaURA3MX4 pan1∆acidic-kanMX6 ABP1-GFP-HIS3 his3∆1 leu2∆0 lys2∆0 met15∆0 ura3∆0* | This study |
| YJC5733 | *MATα las17∆acidic-CaURA3MX4 pan1∆acidic-kanMX6 ABP1-GFP-HIS3 his3∆1 leu2∆0 ura3∆0* | This study |
| YJC5736 | *MATα/MAT****a*** *HIS3MX6-GAL1-GFP-LAS17/LAS17 his3∆1/his3∆1 leu2∆0/leu2∆0 lys2∆0/Lys2 met15∆0/MET15 ura3∆0/ura3∆0* | This study |
| YJC5737 | *MATα/MAT****a*** *HIS3MX6-GAL1-GFP-LAS17/LAS17 his3∆1/his3∆1 leu2∆0/leu2∆0 lys2∆0/Lys2 met15∆0/MET15 ura3∆0/ura3∆0* | This study |
| YJC5756 | *MATα/MAT****a*** *crn1∆::KanMX4/CRN1 ABP1-tdimer2-CaURA3MX4/ABP1 SLA2-GFP-HIS3/SLA2 his3∆1/his3∆1 leu2∆0/leu2∆0 lys2∆0/LYS2 met15∆0/MET15 ura3∆0/ura3∆0* | This study |
| YJC5763 | *MAT****a*** *ABP1-tdimer2-CaURA3MX4 his3∆1 leu2∆0 met15∆0 ura3∆0* | This study |
| YJC5792 | *MATα/MAT****a*** *LAS17-HIS3MX6/LAS17 his3∆1/his3∆1 leu2∆0/leu2∆0 lys2∆0/LYS2 met15∆0/MET15 ura3∆0/ura3∆0* | This study |
| YJC5832 | *MATα/MAT****a*** *URA3-las17-HIS3MX6/LAS17 his3∆1/his3∆1 leu2∆0/leu2∆0 lys2∆0/Lys2 met15∆0/MET15 ura3∆0/ura3∆0* | This study |
| YJC5863 | *MATα/MAT****a*** *ABP1-tdimer2-CaURA3MX4/ABP1 his3∆1/his3∆1 leu2∆0/leu2∆0 lys2∆0/LYS2 met15∆0/MET15 ura3∆0/ura3∆0* | This study |
| YJC5865 | *MATα ABP1-tdimer2-CaURA3MX4 his3∆1 leu2∆0 lys2∆0 MET15 ura3∆0* | This study |
| YJC5866 | *MAT****a*** *HIS3MX6-GAL1-GFP-LAS17 his3∆1 leu2∆0 met15∆0 ura3∆0* | Tis study |
| YJC5868 | *MATα/MAT****a*** *HIS3MX6-GAL1-GFP-LAS17/ LAS17 ABP1-tdimer2-CaURA3MX4/ABP1 his3∆1/his3∆1 leu2∆0/leu2∆0 lys2∆0/LYS2 met15∆0/MET15 ura3∆0/ura3∆0* | This study |
| YJC5872 | *MATα/MAT****a*** *LAS17-GFP-HIS3/LAS17 ABP1-tdimer2-CaURA3MX4/ABP1 his3∆1/his3∆1 leu2∆0/leu2∆0 lys2∆0/LYS2 met15∆0/MET15 ura3∆0/ura3∆0* | This study |
| YJC5950 | *MAT****a*** *LAS17-GFP-HIS3 ABP1-tdimer2-CaURA3MX4 his3∆1 leu2∆0 met15∆0 ura3∆0* | This study |
| YJC5951 | *MATα LAS17-GFP-HIS3 ABP1-tdimer2-CaURA3MX4 his3∆1 leu2∆0 lys2∆0 ura3∆0* | This study |
| YJC5952 | *MAT****a*** *LAS17-GFP-HIS3 ABP1-tdimer2-CaURA3MX4 his3∆1 leu2∆0 ura3∆0* | This study |
| YJC5953 | *MAT****a*** *ABP1-tdimer2-CaURA3MX4 his3∆1 leu2∆0 met15∆0 ura3∆0* | This study |
| YJC5954 | *MATα ABP1-tdimer2-CaURA3MX4 his3∆1 leu2∆0 lys2∆0 ura3∆0* | This study |
| YJC5955 | *MATα ABP1-tdimer2-CaURA3MX4 his3∆1 leu2∆0 ura3∆0* | This study |
| YJC6020 | *MATα/MAT****a*** *GFP-las17-HIS3MX6/LAS17 his3∆1/his3∆1 leu2∆0/leu2∆0 lys2∆0/LYS2 met15∆0/MET15 ura3∆0/ura3∆0* | This study |
| YJC6021 | *MAT****a*** *HIS3MX6-GAL1-GFP-LAS17 ABP1-tdimer2-CaURA3MX4 his3∆1 leu2∆0 met15∆0 ura3∆0* | This study |
| YJC6022 | *MAT****a*** *HIS3MX6-GAL1-GFP-LAS17 ABP1-tdimer2-CaURA3MX4 his3∆1 leu2∆0 met15∆0 ura3∆0* | This study |
| YJC6023 | *MATα ABP1-tdimer2-CaURA3MX4 his3∆1 leu2∆0 lys2∆0 ura3∆0* | This study |
| YJC6024 | *MAT****a*** *ABP1-tdimer2-CaURA3MX4 his3∆1 leu2∆0 lys2∆0 met15∆0 ura3∆0* | This study |
| YJC6025 | *MAT****a*** *ABP1-tdimer2-CaURA3MX4 his3∆1 leu2∆0 lys2∆0 ura3∆0* | This study |
| YJC6039 | *MATα/MAT****a*** *ABP1-tdimer2-CaURA3MX4/ABP1 SLA2-GFP-HIS3/SLA2-GFP las17∆acidic/DDW- CaURA3MX4/LAS17 his3∆1/his3∆1 leu2∆0/leu2∆0 lys2∆0/LYS2 met15∆0/MET15 ura3∆0/ura3∆0* | This study |
| YJC6041 | *MATα ABP1-tdimer2-CaURA3MX4 SLA2-GFP-HIS3 las17∆acidic-CaURA3MX4 his3∆1 leu2∆0 ura3∆0* | This study |
| YJC6043 | *MAT****a*** *ABP1-tdimer2-CaURA3MX4 SLA2-GFP-HIS3 las17∆acidic/DDW- CaURA3MX4 his3∆1 leu2∆0 lys2∆0 met15∆0 ura3∆0* | This study |
| YJC6044 | *MAT****a*** *ABP1-tdimer2-CaURA3MX4 SLA2-GFP-HIS3 his3∆1 leu2∆0 lys2∆0 met15∆0 ura3∆0* | This study |
| YJC6046 | *MATα ABP1-tdimer2-CaURA3MX4 SLA2-GFP-HIS3 his3∆1 leu2∆0 ura3∆0* | This study |
| YJC6178 | *MATα/MAT****a*** *GFP-LAS17-HIS3MX6/LAS17 ABP1-tdimer2-CaURA3MX4/ABP1 his3∆1/his3∆1 leu2∆0/leu2∆0 lys2∆0/LYS2 met15∆0/MET15 ura3∆0/ura3∆0* | This study |
| YJC6179 | *MATα/MAT****a*** *GFP-LAS17-HIS3MX6/LAS17 ABP1-tdimer2-CaURA3MX4/ABP1 his3∆1/his3∆1 leu2∆0/leu2∆0 lys2∆0/LYS2 met15∆0/MET15 ura3∆0/ura3∆0* | This study |
| YJC6180 | *MAT****a*** *ABP1-tdimer2-CaURA3MX4 his3∆1 leu2∆0 lys2∆0 ura3∆0* | This study |
| YJC6181 | *MAT****a*** *ABP1-tdimer2-CaURA3MX4 his3∆1 leu2∆0 ura3∆0* | This study |
| YJC6182 | *MAT****a*** *ABP1-tdimer2-CaURA3MX4 his3∆1 leu2∆0 lys2∆ met15∆ ura3∆0* | This study |
| YJC6183 | *MAT****a*** *GFP-LAS17-HIS3MX6 ABP1-tdimer2-CaURA3MX4 his3∆1 leu2∆0 met15∆0 ura3∆0* | This study |
| YJC6184 | *MATα GFP-LAS17-HIS3MX6 ABP1-tdimer2-CaURA3MX4 his3∆1 leu2∆0 lys2∆0 ura3∆0* | This study |
| YJC6185 | *MATα GFP-LAS17-HIS3MX6 ABP1-tdimer2-CaURA3MX4 his3∆1 leu2∆0 ura3∆0* | This study |
| YJC6187 | *MATα GFP-LAS17-HIS3MX6 his3∆1 leu2∆0 lys2∆0 ura3∆0* | This study |

**Table S1. Yeast strain used in this study.**

1. Brachmann CB, Davies A, Cost GJ, Caputo E, Li J, 0 (1998) Designer deletion strains derived from Saccharomyces cerevisiae S288C: a useful set of strains and plasmids for PCR-mediated gene disruption and other applications. Yeast 14: 115-132.

2. Young ME, Cooper JA, Bridgman PC (2004) Yeast actin patches are networks of branched actin filaments. J Cell Biol 166: 629-635.

3. Kim K, Galletta BJ, Schmidt KO, Chang FS, Blumer KJ, 0 (2006) Actin-based motility during endocytosis in budding yeast. Mol Biol Cell 17: 1354-1363.
